# A152T tau allele causes neurodegeneration that can be ameliorated in a zebrafish model by autophagy induction

**DOI:** 10.1093/brain/awx005

**Published:** 2017-02-09

**Authors:** Ana Lopez, Suzee E. Lee, Kevin Wojta, Eliana Marisa Ramos, Eric Klein, Jason Chen, Adam L. Boxer, Maria Luisa Gorno-Tempini, Daniel H. Geschwind, Lars Schlotawa, Nikolay V. Ogryzko, Eileen H. Bigio, Emily Rogalski, Sandra Weintraub, Marsel M. Mesulam, Tauopathy Genetics Consortium, Angeleen Fleming, Giovanni Coppola, Bruce L. Miller, David C. Rubinsztein

**Affiliations:** 1 Department of Medical Genetics, University of Cambridge, Cambridge Institute for Medical Research, Addenbrooke’s Hospital, Hills Road, Cambridge, CB2 0XY, UK; 2 Department of Physiology, Development and Neuroscience, University of Cambridge, Downing Street, Cambridge CB2 3EG, UK; 3 Memory and Aging Center, Department of Neurology, University of California, San Francisco, CA, USA; 4 Department of Psychiatry and Semel Institute for Neuroscience and Human Behavior, David Geffen School of Medicine, University of California Los Angeles, Los Angeles, CA, USA; 5 Department of Infection, Immunity and Cardiovascular Disease, University of Sheffield, Sheffield, UK; 6 Cognitive Neurology and Alzheimer’s Disease Center, Northwestern University, Chicago, IL 60611, USA

**Keywords:** neurodegeneration, tauopathy, autophagy, proteasome

## Abstract

Mutations in the gene encoding tau (*MAPT*) cause frontotemporal dementia spectrum disorders. A rare tau variant p.A152T was reported as a risk factor for frontotemporal dementia spectrum and Alzheimer’s disease in an initial case-control study. Such findings need replication in an independent cohort. We analysed an independent multinational cohort comprising 3100 patients with neurodegenerative disease and 4351 healthy control subjects and found p.A152T associated with significantly higher risk for clinically defined frontotemporal dementia and progressive supranuclear palsy syndrome. To assess the functional and biochemical consequences of this variant, we generated transgenic zebrafish models expressing wild-type or A152T-tau, where A152T caused neurodegeneration and proteasome compromise. Impaired proteasome activity may also enhance accumulation of other proteins associated with this variant. We increased A152T clearance kinetics by both pharmacological and genetic upregulation of autophagy and ameliorated the disease pathology observed in A152T-tau fish. Thus, autophagy-upregulating therapies may be a strategy for the treatment for tauopathies.

## Teaser

Mutations in *MAPT* cause a variety of neurodegenerative disorders. Lopez *et al.* confirm that A152T-variant tau is associated with increased risk for frontotemporal dementia and progressive supranuclear palsy syndrome. Upregulation of autophagy increases tau clearance and ameliorates pathology in zebrafish expressing A152T-tau, suggesting potential for the treatment of tauopathies.

## Introduction

Neurodegenerative diseases are classified neuropathologically by the distribution and morphology of distinct protein inclusions in the brain. Some diseases share a common pathological protein suggesting potential pathophysiological overlap, but features such as the morphology and anatomical distribution of such inclusions define distinct disease entities. For example, Alzheimer’s disease shows both amyloid-β and microtubule-associated protein tau (MAPT) in the form of neuritic plaques and neurofibrillary tangles, with clinical progression correlating exclusively with neurofibrillary tangle burden (Arriagada *et al.*, 1992). Frontotemporal lobar degeneration (FTLD) shows several disease subtypes of tau pathology, including progressive supranuclear palsy, corticobasal degeneration, and Pick’s disease (Neumann *et al.*, 2009). Both Alzheimer’s disease and FTLD caused by tau (FTLD-tau) feature tau inclusions (Grundke-Iqbal *et al.*, 1986; Hutton *et al.*, 1998; Spillantini *et al.*, 1998); however, other forms of presenile dementia with neurofibrillary lesions but no amyloid-β deposits have also been described (Sumi *et al.*, 1992; Spillantini *et al.*, 1996; Goedert, 2016), suggesting that neurofibrillary tangles frequently observed in early stages of Alzheimer’s disease pathology can occur independently of amyloid-β deposits and are associated with dementia.

Although the known *MAPT* mutations appear to exclusively cause FTLD-tau pathology, the rare p.A152T variant, first identified in a patient with dementia with behavioural symptoms and unclassifiable tau pathology (Kovacs *et al.*, 2011), was identified as a genetic risk factor for both clinically-defined Alzheimer’s disease and frontotemporal dementia (FTD) spectrum disorders in an initial genetic association study (Coppola *et al.*, 2012). p.A152T is a single G > A nucleotide change in a non-microtubule-binding region of exon 7 in *MAPT*, and diminishes tau binding to microtubules, while increasing tau oligomer formation (Coppola *et al.*, 2012). p.A152T carriers with neurodegenerative disease show heterogeneous clinical syndromes, including progressive supranuclear palsy syndrome (PSP-S), behavioural variant FTD, non-fluent variant primary progressive aphasia, corticobasal syndrome, clinical Alzheimer’s disease, and dementia with Lewy bodies (Lee *et al.*, 2013*b*; Labbe *et al.*, 2015). The term ‘syndrome’ is used because the underlying neuropathology for these disorders is variable, particularly corticobasal syndrome. Together, these studies suggest that p.A152T is a risk factor for diverse neurodegenerative disease syndromes related to FTLD-tau and possibly synucleinopathy, although recently, a study of 11 572 subjects in Spain did not observe an increased association between p.A152T and Alzheimer’s disease, while the association with FTD spectrum approached significance [odds ratio (OR) = 2.03; *P = *0.063] (Pastor *et al.*, 2016).

To resolve whether p.A152T is a risk variant, we performed a replication study to screen the p.A152T variant in an independent multinational cohort consisting of 3100 patients with neurodegenerative disease and 4351 healthy control subjects. Furthermore, to assess whether the A152T-tau showed greater toxicity than wild-type-tau *in vivo*, we generated new zebrafish models expressing either wild-type or A152T human tau, which revealed more neurodegeneration and tau pathology in the disease-allele associated variant fish. Previously we found that wild-type forms of tau, like many other intracytoplasmic aggregate-prone proteins, can be degraded by macroautophagy (henceforth autophagy), as well as via the ubiquitin–proteasome pathway (Berger *et al.*, 2006; Moreau *et al.*, 2014; reviewed in Lee *et al.*, 2013*a*). Autophagy involves the sequestration of portions of cytoplasm into double-membrane vesicles called autophagosomes, which are then trafficked to lysosomes that mediate the degradation of the autophagosomes and their contents (Rubinsztein, 2006; Rubinsztein *et al.*, 2012). We found the A152T variant to be associated with disruption of proteasome function and delayed tau clearance *in vivo*, whereas autophagy function was unaffected. Hence, we assessed the potential utility of autophagy upregulation to improve clearance, and found that this could enhance the degradation of A152T-tau in zebrafish and ameliorate its toxicity, suggesting a possible therapeutic strategy for these tauopathies.

## Materials and methods

### Participants

We included 3100 patients with neurodegenerative disease and 4351 controls recruited worldwide across collaborating centres, including: (i) University of California San Francisco and the multicentre Davunetide trial (Boxer *et al.*, 2014) (Allon series); (ii) Gladstone Institute (Gladstone Turkish series); (iii) University of Brescia and San Raffaele Scientific Institute (Italian series); (iv) Northwestern University (Northwestern series); (v) Rush Alzheimer’s Disease Center (Religious Orders Study and Rush Memory and Aging Project); (vi) University of California Los Angeles (Small series); (vii) Instituto de Salud Carlos III, Madrid, Spain (Spanish series); (viii) University of Toronto Memory Clinic (Toronto series); (ix) School of Medicine, Yale University (Turkish series); (x) University of California San Francisco Memory and Aging Center (UCSF series); and (xi) other centres (Other series). All individuals gave authorization for genetic testing research in accordance with the local regulations. Each institution’s Committee on Human Research approved the study. Clinical diagnoses were rendered by expert neurologists at each institution. In this study, a diagnosis of FTD denotes clinically defined behavioural variant FTD or primary progressive aphasia. Participants or their surrogates provided informed consent before participation.

### Genetic analysis

Genotypes were obtained using TaqMan® SNP assays from Life Technologies on a LightCycler® 480 System. A custom assay (#AHHR7R6) was designed for *MAPT* p.A152T rs143624519. Forward primer sequence was CCAATGGTGAAAAACCCCTCTATCA and reverse primer sequence was TTGGCCTGGCCCTTCTG. Reporter sequences were AAAACGAAGATCACCACACC and ACGAAGATCGCCACACC. p.A152T carriers were confirmed using Sanger sequencing. Statistical analysis was performed in R (version 3.1.3, www.r-project.org).

### Zebrafish experiments

#### Maintenance of stocks and collection of embryos

All zebrafish experiments were performed in accordance with Home Office Guidelines and local ethical committee approval. Zebrafish were reared under standard conditions on a 14 h light: 10 h dark cycle. Embryos were collected from natural spawnings, staged according to established criteria (Kimmel *et al.*, 1995) and reared in embryo medium (5 mM NaCl, 0.17 mM KCl, 0.33 mMCaCl_2_, 0.33 mM Mg_2_SO_4,_ 5 mM HEPES) at 28.5°C in the dark. The EIF1α::Gal4VP16 line was made in-house by subcloning the *Xenopus* EIF1α promoter (Burket *et al.*, 2008) into a 5’ entry cloning vector (Multisite Gateway® technology, ThermoFisher), and recombination was performed according to the manufacturer’s instructions using pME-Gal4VP16 and p3E-polyA components of the Tol2kit (Kwan *et al.*, 2007). The beta-actin::Gal4VP16 line was generated from a transgenic construct comprising p5E-beta-actin, pME-Gal4-VP16 and p3E-polyA, components of the Tol2kit, using Gateway® recombination (Life Technologies) into the destination vector pDestTol2CryECFP. pDestTol2CryECFP was generated by cloning a Cry:ECFP expression cassette (a kind gift of Michael Parsons) into the destination vector pDestTol2CG2 (Kwan *et al.*, 2007) to replace the CG2 transgenic marker. The pan-neuronal Gal4 driver line (hereafter referred to as PanN::Gal4VP16) was a kind gift from Herwig Baier [identified as line s1101tEt in the original publication (Scott *et al.*, 2007)].

#### Generation and microinjection of Dendra-tau constructs

Human wild-type and A152T mutant *MAPT* (2N4R) in pCDNA3.1 (a gift from Dr Li Gan) were subcloned into pDendra2 (Adam *et al.*, 2009) at KpnI and ApaI sites. The Dendra-tau fusion construct was then subcloned into a middle entry cloning vector (Multisite Gateway® technology, ThermoFisher), and recombination was performed according to the manufacturer’s instructions using p5E-UAS and p3E-polyA components of the Tol2kit (Kwan *et al.*, 2007) to generate the transgenic construct UAS::Dendra-tau-polyA within a destination vector (pDestTol2CG2). The destination vector contains a reporter construct [comprising EGFP driven by the cardiac myosin light chain (CMLC) promoter in the reverse orientation to the Dendra-tau transgene] and is flanked with Tol2 sites to facilitate genomic integration. The resulting vectors for Dendra-tauWT and Dendra-tauA152T were termed Responder constructs (Supplementary Fig. 1). Plasmids were prepared by extraction with a NucleoBond® Xtra plasmid purification Kit (Machery Nagel) and subsequent phenol/chloroform extraction. *Tol2* transposase mRNA was synthesized from the Not1-linearized pCS2FA-transposase vector (Kwan *et al.*, 2007) with the Ambion mMESSAGE mMACHINE® kit (Applied Biosystems) according to the manufacturer’s instructions.

Responder constructs were mixed with *Tol2* transposase mRNA, at a final concentration of 100 ng/μl of DNA and 25 ng/μl of RNA in Danieau’s solution (58 mM NaCl, 0.7 mM KCl, 0.4 mM MgSO_4_.7H_2_O, 0.6 mM, 5 mM HEPES, pH 7.6) containing 20% phenol red. The freshly prepared DNA/mRNA was injected into the cell of 1 cell-stage embryos of the EIF1α::Gal4VP16 transgenic line. At 3 days post-fertilization (dpf), embryos were selected for mosaic expression of Dendra-tau using EGFP filter sets on an Olympus SZX12 stereo fluorescence microscope then raised to adulthood. Adult F0 mosaic fish were outcrossed to TL wild-type fish and the F1 generation was screened to identify embryos with green hearts (EGFP driven from the CMLC::EGFP reporter) to identify germ line transmitting founders without EIF1α::Gal4VP16 background. These embryos were raised to establish responder transgenic lines (Supplementary Fig. 1).

#### Experimental crosses

The eggs collected from a single cross at a single time are generically termed as a clutch. Crosses with EIF1α::Gal4VP16 driver fish and responder fish resulted in ubiquitous but mosaic expression of Dendra-tau, which were used for clearance assays. Crosses with beta-actin::Gal4VP16 driver fish and responder fish were used for analysis of proteasome function. Crosses between PanN::Gal4VP16 driver fish and responder fish resulted in Dendra-tau expressed throughout the CNS in a similar distribution to the expression of endogenous tau (Chen *et al.*, 2009); such crosses were used for all other experiments (Supplementary Fig. 1).

#### Fluorescence and immunofluorescence microscopy

Whole-mount antibody staining was performed according to standard methods (Schulte-Merker, 2002) using the following concentrations of antibodies: 1:100 mouse anti-AT8 (Pierce, Thermo Scientific); 1:500 mouse anti-α-acetylated tubulin antibody (Sigma-Aldrich); Alexa Fluor® 568 (Invitrogen). Samples for phosphorylation analysis were mounted in 3% methyl cellulose and imaged using a Zeiss Axio Zoom.V16 microscope with Apotome system. Images comparing two groups of fish were performed at the same time using identical settings. Antibody staining for alpha-acetylated tubulin was performed on fish at 3 dpf to quantify abnormalities in motor neuron length and bifurcation (*n = *18 fish/group) as explained in Fig. 2B using a GX Optical LED fluorescent microscope, GXCAM3.3 digital camera and GX Capture software. Antibody staining of cryosections was performed to detect phosphorylated tau at residues Thr181 (AT270, 1:50; Pierce, Thermo Scientific), Ser202/Thr205 (AT8; 1:50; Pierce, Thermo Scientific) and Ser396/Ser404 (PHF1, 1:100; a kind gift from Dr Peter Davies, Albert Einstein College of Medicine of Yeshiva University, NY); the conformational tau marker MC1 (1:50; a kind gift from Dr Peter Davies, Albert Einstein College of Medicine of Yeshiva University, NY); and cholinergic neurons (anti-ChAT, 1:125, goat polyclonal anti-ChAT antibody from Chemicon International). Ten micrometre cryosections were cut using a Bright cryostat, incubated in 2% H_2_O_2_/phosphate-buffered saline (PBS) for 20 min to reduce background staining, blocked in 10% normal goat or donkey serum (NGS, NDS respectively) in PBS 0.1% Tween-20 (PBST) for 1 h at room temperature and incubated with primary antibody in PBST with 0.2% Tween-20 and 10% NGS or NDS overnight at 4°C (for 2 days in case of ChAT detection). Sections were washed, incubated with Alexa Fluor® 568 secondary antibodies then washed and mounted using VECTASHIELD® HardSet with DAPI (Vectorlabs) and visualized using a Zeiss Axio Zoom.V16 microscope. Cholinergic neuron number was quantified from transverse sections of spinal cord of larvae at 6 dpf. The number of ChAT-positive motor neurons was quantified manually across 20 sections of the spinal cord from the dorsal fin region (*n = *5 fish/group).

Phenotypic analysis was performed using fluorescence and brightfield microscopy on a Zeiss Axio Zoom.V16 microscope. Intensity of fluorescence in whole fish was analysed using the ‘ROI manager’ tool in ImageJ software. Analysis of axonal defects was performed on live transgenic larvae at 3 dpf. Larvae were anaesthetized with tricaine, flat mounted in 1% low melting agarose in embryo medium and viewed using a Leica SP2 laser confocal microscope using a 20 × objective and ‘z-stack’ tool. Images were then processed with FIJI software (ImageJ) with maximum projections of *z*-stacks with constant stack height containing the whole neuronal projection.

#### RNA preparation and quantitative reverse transcription polymerase chain reaction

Pools as well as individual embryos from independent clutches of WT-tau and A152T-tau fish crossed to driver PanN::Gal4VP16 fish were collected at 24 hours post-fertilization (hpf) (pre-phenotype) and 3 dpf (post-phenotype). Some siblings from each clutch were set aside and kept for subsequent quantification of phenotypic defects at 3 dpf (post-phenotype). Total RNA from a pool of *n = *10 fish from the same clutch or from single fish was isolated using RNeasy-Plus Mini-Kit® (Qiagen) according to manufacturer’s instructions. A total of 50 ng of RNA from pools of fish and 10 ng from individual fish was then used in One-Step qRT-PCR combining cDNA synthesis by specific-primed reverse transcription and real-time PCR reaction according to manufacturer protocols (Invitrogen) using TaqMan® Enzyme mix and customized TaqMan® gene-specific primers for Gal4, Dendra and *GAPDH* as housekeeping gene (GAPDH TaqMan® made-to-order gene expression code 4351372 Dr03436845_g1 from Applied Biosystem; Gal4_F GCTCAAGTGCTCCAAAGAAAAACC and GAL4_R CGACACTCCCAGTTGTTCTTCA; Dendra_F ACAAGGGCATCTGCACCAT and Dendra_R AAGCGCACGTTCTGGAAGA). All samples were run in triplicate and were analysed on a StepOne Plus Real Time PCR System and StepOne^TM^ Sofware V.2.1 (Applied Biosystems, Life Technologies). Relative gene expression was normalized to *GAPDH* controls and assessed using the 2−ΔΔCT method.

#### Western blotting

Fish were collected and lysed on ice with lysis buffer containing 1% octylglucoside, complete protease inhibitor cocktail and PhosSTOP^™^ tablets (Sigma). Fish were homogenized by sonication and lysates were centrifuged at 7000 rpm for 1 min at 4°C. Supernatants were diluted in 2 × Laemmli Buffer at a 1:1 dilution, resolved by sodium dodecyl sulphate polyacrylamide gel electrophoresis (12% and 16% gels) and transferred to PVDF membranes. The membranes were blocked with PBST containing 5% non-fat dry milk and were then incubated overnight at 4°C with primary antibodies diluted in PBST. Membranes were washed three times in PBST, incubated for 1 h at room temperature with 1:5000 dilution of horseradish peroxidase-conjugated secondary anti-mouse or anti-rabbit antibodies (Dako) in PBST, and washed three times for 10 min each. Immunoreactive bands were then detected using ECL^™^ (GE Healthcare Bioscience). Quantification of proteins normalized to actin, GAPDH or Dendra was performed using ImageJ (FIJI) software. The following antibodies were used: mouse anti-Actin (1:1000; Sigma-Aldrich), mouse anti-GAPDH (1:1000; Millipore), rabbit anti-Dendra (1:1000; Online Antibodies), Tau5 mouse anti-tau (1:1000; Abcam), mouse anti-AT270 (1:1000; Pierce, Thermo Scientific), mouse anti-AT8 (1:200; Pierce, Thermo Scientific), mouse anti-PHF1 (1:100; a kind gift from Dr Peter Davies, Albert Einstein College of Medicine of Yeshiva University, NY) rabbit antibody against cleaved (activated) caspase 3 (1:100; Abcam), rabbit anti-Atg5 antibody (1:1000; Abcam) and rabbit anti-LC3 antibody (1:1000; Novus Biologicals) mouse anti-α1-7 subunits of the proteasome 20S (1:1000; Enzo), mouse anti-P4D1 antibody for ubiquitinated proteins (1:1000, Cell Signalling), rabbit anti-GFP antibody (1:1000, Abcam).

#### TUNEL

Terminal deoxynucleotidyl transferase dUTP nick end labelling (TUNEL) assay was performed using an In Situ Cell Death detection kit (Fluorescein or TMR; Roche Diagnostics). Ten micrometre transverse or longitudinal cryosections were fixed in 4% paraformaldehyde at room temperature for 15 min and permeabilized with 20 µg/ml proteinase K. A slide treated with DNase (4 U/200 µl; Ambion) was used as positive control. TUNEL was performed according to the manufacturer’s instructions and sections were mounted with VECTASHIELD® Hard Set containing DAPI (Vectorlabs). Digital images were captured using GX Optical LED fluorescent microscope and GXCAM3.3 digital camera using GX Capture software (Version 6.2.3.0). Longitudinal sections of Dendra-tau fish from 24 hpf to 5 dpf were used in a first approach to determine a suitable time-point for the quantification of cell death. Transverse sections through the brain of 2 dpf and 6 dpf zebrafish larvae were used to quantify the number of apoptotic neurons. The mean number of TUNEL-positive nuclei was calculated for each genotype and phenotype (*n = *5 fish) from a minimum of five sections across the brain and eyes per fish.

#### Isolation of soluble and sarkosyl-insoluble tau fractions

Fractionation and analysis of soluble and insoluble tau was performed as previously described (Santacruz *et al.*, 2005) with minor modifications. WT-tau and A152T-tau fish were collected dry at 24 hpf and 3 dpf and stored at −80°C (55 fish collected per independent sample, corresponding to ∼18 mg). Frozen samples were homogenized by addition of 10 v/w ice-cold Tris-buffered saline (TBS: 25 mM Tris pH 7.4, 150 mM NaCl, 1 mM EDTA, 1 mM EGTA) supplemented with Protease Inhibitor Cocktail, 1 mM PMSF, phosphatase inhibitor cocktail II and III (all Sigma) and disrupted using glass beads in a Tissuelyser LT (Qiagen). Sample homogenate (150 μl) was ultracentrifuged at 150 000 *g* for 15 min at 4°C. Supernatant was collected (soluble tau fraction) and protein quantification performed. The pellet was re-homogenized in 150 μl high salt/sucrose buffer (10 mM Tris pH7.4, 0.8 M NaCl, 10% Sucrose, 1 mM EGTA, 1 mM PMSF) and ultracentifuged as before. Supernatant was incubated with a final volume of 1% sarkosyl for 1 h at 37°C before ultracentrifugation at 150 000 *g* for 1 h at 4°C. The pellet (sarkosyl-insoluble tau) was resuspended directly in 30 μl of Laemmli buffer and boiled at 100°C for 5 min. Western blots were performed as previously described using soluble and sarkosyl-insoluble tau samples run on 10% tris-glycine gels. Blots were blocked in 5% non-fat milk in PBST and incubated with primary antibodies Tau-5 (1:1000, Abcam) and actin (1:1000 Sigma-Aldrich).

#### Thioflavin-S staining

Modified Thioflavin-S staining (Guntern *et al.*, 1992) was performed on transverse cryosections of 6 dpf larvae at the position of the optic chiasm. The sections were pretreated in potassium permanganate solution for 4 min followed by bleach treatment, then rinsed in water and incubated in 0.1% Thioflavin-S in 50% ethanol for 5 min at room temperature in the dark. Sections were briefly differentiated in 50% ethanol solution, rinsed in water then mounted using VECTASHIELD® Hard Set with DAPI. Sections were examined using a Zeiss Axioplan2 fluorescent microscope equipped with a QImaging Retiga 2000 R digital camera.

#### Quantification of Dendra-tau clearance

Crosses of UAS::Dendra-tau responder fish with EIF1α::Gal4VP16 driver fish were performed to produce offspring with ubiquitous but mosaic expression of the transgene to allow visualization of individual neurons in the spinal cord. Embryos were sorted at 24 hpf to identify fluorescent Dendra-tau expressing individuals. Photoconversion of individual spinal cord neurons was performed on 2 dpf larvae anaesthetized by immersion in 0.2 mg/ml 3-amino benzoic acid ethyl ester (MS222) and mounted in 1% low melting agarose in embryo medium. Photoconversion of Dendra, previously described to track protein dynamics (Chudakov *et al.*, 2007), was performed by UV irradiation (405 nm) for 3 s using Bleachpoint tool and a confocal microscope Leica SP8 at ×3 magnification with 40× objective. Digital fluorescent images of individual neurons in larvae with Dendra-tau mosaic expression were taken immediately after photoconversion and at 12-h intervals over a further 48 h. The images were then analysed using ImageJ by selecting regions of interest around each neuron with Dendra-tau expression and fluorescent intensity was measured using ‘ROI’ and ‘Integrated Density’ functions. To monitor Dendra-tau clearance the fluorescent intensity of each cell was quantified at each time point and expressed as a percentage of the initial fluorescent intensity, immediately after photoconversion.

#### Compound treatment in Dendra-tau fish

Dendra-tau embryos were reared in embryo medium until 24 hpf then treated with either 0.1% dimethyl sulphoxide (DMSO), 30 μM rapamycin, 50 µM rilmenidine or 30 µM clonidine from 24 hpf to 3 dpf with drugs and medium replenished daily. The percentage of larvae with different phenotypes was quantified at 3 dpf. Phenotypes were classified either as normal; mild (when slight torsion of dorsal spine was observed); moderate (when head axis was not aligned to the dorsal spine but the larvae could swim straight); and severe (when fish showed a complete torsion of whole body in ‘U’-shape). To analyse the clearance of Dendra-tau in presence of modulators of autophagy or proteasome function, fish were reared in conventional embryo medium until the moment of photoconversion at 48 hpf and immediately after treated with 0.1% DMSO, 30 µM clonidine, 30 μM rapamycin, 50 μM rilmenidine, 10 mM NH_4_Cl or 100 µM MG132, also refreshed daily.

#### Behavioural assays

Zebrafish larvae (72 hpf and 6 dpf) from independent clutches were sorted for fluorescent Dendra-tau expression and tested individually. The WT-tau and A152T-tau fish were compared to negative (non-fluorescent) siblings. Motility was analysed by tapping the dish containing a single fish to induce the escape response. The gentle tap was repeated three times with a rest time of 15 s inbetween to avoid habituation. Only the movements involving motion to a different position were considered an escape response. Three replicate experiments were performed with a minimum of 20 fish per genotype per experiment using larvae from independent clutches for each replicate.

#### Atg5 injection

A middle entry clone vector (Multisite Gateway® technology, ThermoFisher) containing Zebrafish *atg5* was generated after adding a 5’ attB1 site followed by a FLAG tag and a 3’ attB2 site by PCR (attB1FLAGatg5fow 5’-GGGGACAAGTTTGTACAAAAAAGCAGGCTCAATGGACTACAAAGACGATGACGACAAGATGATAATGGCAGATGACAAG-3’ attB2atg5rev 5’-GGGGACCACTTTGTACAAGAAAGCTGGGTAGAGAGCGGGAGGGTTAATGT-3’) using an *atg5* cDNA clone (Source Bioscience, MGC 100934, IMAGE:7145909) as template. Recombination of the PCR product and the pDONR221 vector clone was performed according to the manufacturer’s instructions. The transgenic construct UAS::FLAGatg5-polyA was generated using the pME_FLAGatg5 vector together with p5E-UAS and p3E-polyA components of the Tol2 kit within a destination vector (pDestTol2CG2) (Kwan *et al.*, 2007). The destination vector contains a reporter construct (comprising EGFP driven by the CMLC promoter in the reverse orientation to the *atg5* transgene) and is flanked with *Tol2* sites to facilitate genomic integration.

The UAS construct containing *atg5* was co-injected with *Tol2* transposase mRNA (25 ng/μl of DNA and 25 ng/μl of RNA in Danieau’s solution containing 20% phenol red) into the cell of 1 cell-stage embryos from the cross of A152T-tau responder fish with PanN::Gal4VP16 driver fish. At 2 dpf, injected embryos and non-injected siblings expressing Dendra-tau were analysed to quantify the proportion of phenotypes, then collected for western blotting as previously described.

#### Proteasome function assay

Dendra-tau positive larvae (48 hpf) from at least six independent clutches of PanN::Gal4VP16 crossed with responder fish were homogenized in 50 mM Tris (pH 7.5), 1 mM DTT by brief sonication (twice for 10 s) and each clutch was analysed in triplicate for control (1% DMSO) and MG132 (1.5 μM) treatments. Tissue lysates were centrifuged at 16 900 *g* for 10 min at 4°C, protein concentration determined using a fluorescence-based quantitation assay kit (Qubit®, ThermoFisher Scientific). Samples normalized for protein concentration (20 μg total amount of protein) were loaded in a 96-well-plate. Chymotrypsin-like activity of the 26S proteasome was monitored using a FLUOstar Omega fluorometer (BMG Labtech) with Omega.LNK software. The reaction started by the addition 100 μM Suc-LLVY-AMC (Enzo) diluted in homogenizing buffer (‘time zero’). The activity was assayed by measuring the fluorescence intensity during 3 h at 37°C, in 5-min intervals (excitation 355 nm; emission 460 nm). Samples of the same lysates were also loaded on a 16% non-denaturing gel for western blot analysis to detect proteasome 20S core particle levels using the anti-α1-7 antibody.

#### Ub^G76V^-GFP injections

The proteasome reporter Ub^G76V^-GFP in pEGFP-N1 [kindly provided by N. Dantuma (Dantuma *et al.*, 2000)] was subcloned into a middle entry cloning vector (Multisite Gateway® technology, ThermoFisher) at NheI and NotI sites and recombination was performed with p5E-UAS and p3E-polyA into pDestTol2CG2 (components of the Tol2kit; Kwan *et al.*, 2007) to generate the UAS::Ub^G76V^-GFP reporter construct flanked with *Tol2* sites to facilitate genomic integration. The UAS::Ub^G76V^-GFP reporter construct was co-injected with the *Tol2* transposase mRNA (25 ng/μl of DNA and 25 ng/μl of mRNA in Danieau’s solution containing 20% phenol red) into the cell of one cell-stage embryos from the cross of A152T-tau responder fish with beta-actin::Gal4VP16 driver fish. At 24 hpf embryos were selected for Dendra-tau expression and treated either with DMSO or 100 μM MG132. After 8 h of treatment, fish were anaesthetized and collected for western blotting as described previously.

### Statistics

Mean values, standard error of the mean (SEM) and standard deviations were calculated with Microsoft Excel. Statistical analysis was performed using two-tailed Student’s *t*-test or Student-Newman-Keuls one-way ANOVA in GraphPad InStat 3. Data are presented as mean ± SEM or standard deviation (SD). *P* < 0.05 was considered significant.

## Results

### p.A152T shows significantly higher risk for progressive supranuclear palsy syndrome and frontotemporal dementia

**Figure 1:**
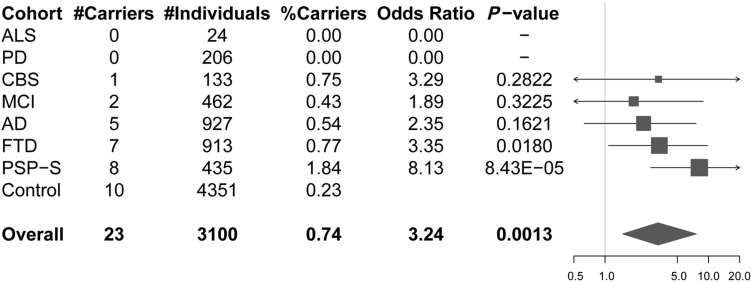
***MAPT* p.A152T carrier frequencies and associated odds ratio for the different disease cohorts.** The total number of individuals and p.A152T carriers for each of the disease cohorts and controls is shown in the table (*left*), with odds ratios and nominal *P*-values depicted in the forest plot (*right*). Overall refers to the combined neurological disease patient samples. In the forest plot, squares represent the estimate odds ratio and are drawn proportional to the weight of the sample and lines represent 95% confidence intervals.

Among 3100 patients with neurodegenerative disease, we identified 23 p.A152T carriers (Fig. 1): one carrier in 133 cases with corticobasal syndrome (0.75%); two carriers in 462 cases with mild cognitive impairment (0.43%); five carriers in 927 cases with Alzheimer’s disease (0.54%); seven carriers in 913 FTD cases (0.77%); and eight in 435 cases with PSP-S (1.84%). No carrier was identified among our cohorts of amyotrophic lateral sclerosis (*n = *24) and Parkinson’s disease (*n = *206) individuals. Ten in 4351 control individuals carried the p.A152T allele (0.23%). This frequency is similar to the one observed in over 60 000 unrelated individuals from the Exome Aggregation Consortium (ExAC, http://exac.broadinstitute.org/), with 159 carriers (three of them homozygous) among 60 472 individuals (0.26%). Demographic characteristics of this replication cohort are described in Supplementary Table 1.

A combined analysis performed on the 3100 patients placed the estimated OR at 3.24 (CI: 1.48–7.65, Fisher’s exact test, *P = *0.0013) for overall neurodegenerative diseases versus controls. Analyses on the different individual disease cohorts, revealed OR ranging between 1.89 (in the MCI cohort) and 8.13 (in the PSP-S cohort) (Fig. 1). However, the association was only significant for the PSP-S (OR = 8.13, CI: 2.77–22.99, Fisher’s exact test, *P = *8.43 × 10^−5^) cohort and nominally significant for the FTD (OR = 3.35, CI: 1.08–9.79, Fisher’s exact test, *P = *0.0180) cohort. As this is a targeted replication study aiming to test a single variant, these *P*-values are sufficient support for the hypothesis, as they do not require correction for testing of multiple loci.

### A152T-tau zebrafish show abnormal phenotypes and motility defects

**Figure 2:**
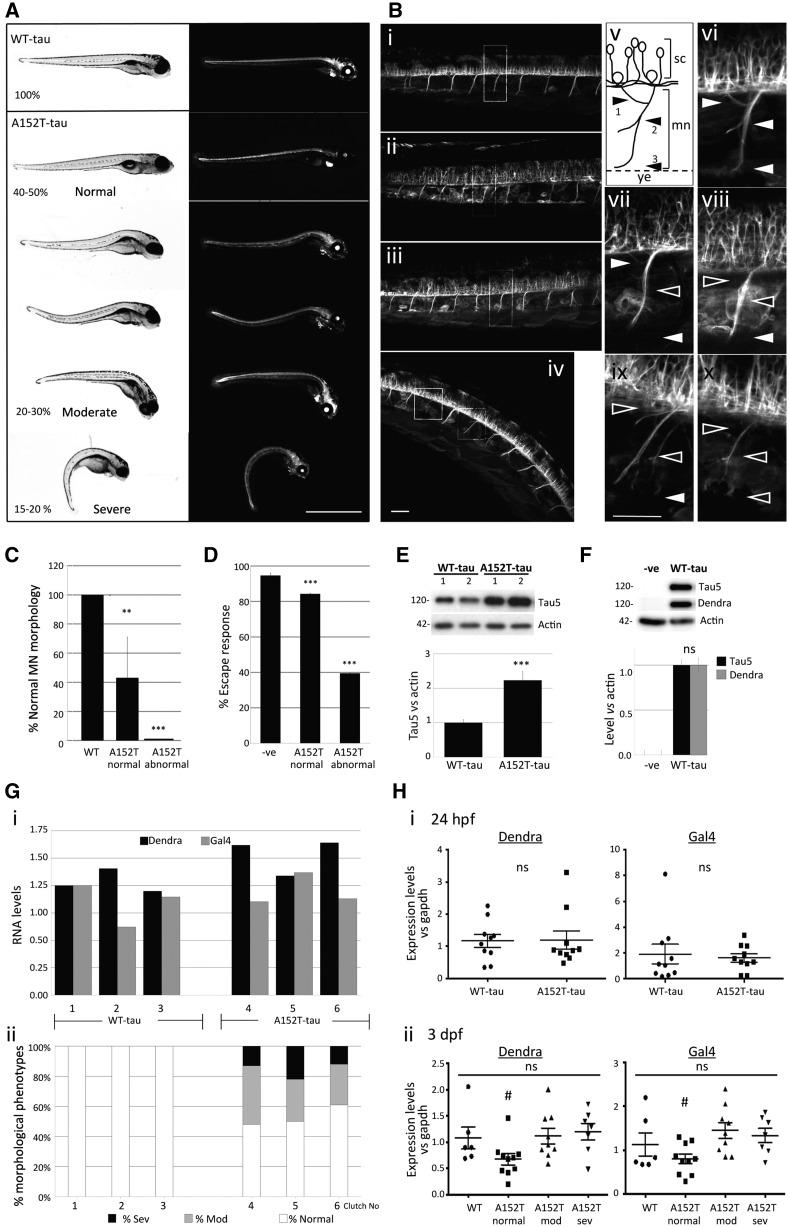
**Phenotypic characterization of Dendra-tau transgenic zebrafish.** (**A**) Representative images of fish with pan-neuronal expression of WT-tau and A152T-tau. In all clutches of WT-tau offspring, larvae were normal. In contrast, A152T-tau fish showed abnormal phenotypes in ∼50% of each clutch. Differing degrees of abnormal curvature of the dorsal spine was observed in A152T-tau fish (percentages refer to the number of the larvae within each severity range per clutch; observations based on more than 30 individual clutches per transgenic line). Scale bar = 1 mm. (**B**) Motor neuron morphology was analysed by confocal microscopy on live fish and abnormalities found only in A152T-tau fish including truncation, abnormal pathfinding and aberrant branching (ramifications) (**i**) WT-tau; (**ii–iv**) A152T-tau, with normal (**ii**), moderate (**iii**) and severe (**iv**) phenotypes. (**v**) Schematic view of a single somatic motor neuron (mn) unit from the spinal cord (sc) to the yolk extension (ye) and its ramifications divided into dorsal (arrowhead 1) and medial (arrowhead 2). Arrowhead 3 labels normal axonal length reaching the yolk extension. Panels **vi–x** correspond to magnified images of **i–iv** highlighting normal (white arrowheads) or abnormal (black arrowheads) ramifications and length according to axonal scheme represented in **v** [**vi** corresponds to WT-tau (**i**); **vii** corresponds to normal phenotype A152T-tau (i**i**); **viii** corresponds to moderate phenotype A152T-tau (**iii**); **ix** and **x** correspond to severe phenotype A152T-tau (**iv**)]. Scale bar = 50 µm. See also Supplementary Fig. 2. (**C**) Quantification of the branching defects observed in motor neurons (MN) according to scheme in **B**(**v**) at 3 dpf. Abnormalities at any specified point (arrowheads 1, 2 or 3) were counted as abnormal (five segmental motor neuron units within the yolk sac extension region of the trunk, anterior to the urogenic opening, were counted for *n = *18 fish per group; graph represents mean ± SD; two-tailed *t*-test, ^**^P < 0.01 and ^***^P < 0.001 versus WT-tau). (**D**) Escape response defects were observed in A152T-tau but not WT-tau fish at 6 dpf (three independent experiments in triplicate, *n = *20/group shown as mean ± standard error; ^***^P < 0.001 versus negative siblings by two-tailed *t*-test). (**E**) Fish expressing A152T-tau show significantly higher levels of total human tau protein at 3 dpf (mean ± SEM, *n = *3 independent clutches in triplicate, ^***^P < 0.001 versus WT-tau by two-tailed *t*-test). (**F**) Levels of Dendra-tau protein (∼120 kDa) could be equally detected by western blot with either Dendra or Tau5 antibodies (mean ± standard error of four clutches in triplicate, by two-tailed *t*-test). (**G**) The higher levels of Dendra-tau together with the morphological and motility defects observed in A152T-tau fish are not the result of higher levels of expression of the transgene (mRNA levels). [**G**(**i**)] Quantification of the mRNA expression levels of Dendra and Gal4 by quantitative RT-PCR at 24 hpf (pre-phenotype) shows variability in the expression of Dendra between different clutches of WT-tau fish (three independent clutches labelled 1–3) and A152T-tau fish (three independent clutches labelled 4–6). Analysis was performed on groups containing *n = *10 fish. [**G**(**ii**)] Phenotypic assessment of larvae at 3.dpf from the same clutches analysed in [**G**(**i**)] shows abnormal phenotypes in all clutches of A152T-tau fish regardless of the expression level of Dendra-tau. The similar levels of expression of Dendra in clutches 2 (WT-tau) and 5 (A152T-tau) at 24 hpf resulted in abnormal phenotypes only in A152T-tau expressing fish (sev = severe, mod = moderate, according to morphological phenotype score presented in **A**). (**H**) Quantification of the mRNA expression levels of Dendra and Gal4 from 10 individual fish collected from clutches 2 (WT-tau) and 5 (A152T-tau). When siblings from these clutches were analysed as pooled samples, equal Dendra mRNA expression levels were observed at 24 hpf. When mRNA expression levels of Dendra or Gal4 were measured in single WT-tau and A152T-tau fish at 24 hpf (pre-phenotype), no significant differences were observed. [**H**(**i**)]. At 3 dpf [**H**(**ii**)] WT-tau and A152T-tau individual fish with moderate and severe phenotypes had equivalent levels of Dendra and Gal4 mRNA expression. However, A152T-tau fish which were morphologically normal were found to have significantly lower levels of Dendra mRNA expression [**H**(**ii**)].

To test the functional consequences of the A152T variant *in vivo*, we generated new zebrafish models exploiting the Gal4-UAS binary expression system (Halpern *et al.*, 2008). We used responder constructs comprising wild-type or mutant A152T human tau (2N4R) fused to the sequence encoding the photoactivatable protein Dendra (Adam *et al.*, 2009), downstream of UAS. Dendra-tau expression was driven either ubiquitously using EIF1α::GAL4-VP16 or beta-actin::Gal4VP16, or throughout the nervous system using pan-neuronal::GAL4-VP16 (PanN::Gal4VP16) drivers (Supplementary Fig. 1A–C). Pan-neuronal expression of the wild-type tau transgene (WT-tau) caused no obvious morphological defects, whereas fish expressing A152T mutant tau (A152T-tau) showed abnormal phenotypes in 50% of the clutch, the most significant feature being abnormal curvature of the dorsal spine (Fig. 2A). Analysis of the motor neurons revealed no abnormalities in the WT-tau transgenic fish, while pathfinding and branching defects were observed in A152T-tau fish, notably even in fish with normal gross morphology (Fig. 2B and C and Supplementary Fig. 2A). In addition, the motor neuron population in the spinal cord was markedly reduced in A152T-tau at fish at 6 dpf (Supplementary Fig. 2B and C). In accordance with these neuronal defects, A152T-tau larvae had impaired escape responses to stimuli (Fig. 2D and Supplementary Fig. 2D).

### Wild-type and mutant transgenes are expressed at similar levels but tau protein levels are increased in A152T-tau fish

As morphological and behavioural defects were only observed in A152T-tau fish, we next assessed whether this was a consequence of different levels of transgene expression. At 3 dpf, Dendra-tau protein was observed to be significantly higher in A152T-tau fish compared to those expressing the WT-tau (Fig. 2E). We confirmed that this was specifically expression of the Dendra-tau fusion protein as the same band could be detected with both Dendra and Tau5 antibodies (the latter raised against human tau protein; Fig. 2F and Supplementary Fig. 4D). However, we also observed that the fluorescent Dendra signal (which is a surrogate for tau protein levels, as this is a fusion protein), was not different between the two transgenic lines at the onset of transgene expression at 24 hpf (Supplementary Fig. 3A). Similarly, when larvae were selected for similar expression levels of Dendra fluorescence at 24 hpf (pre-phenotype), fish expressing the WT-tau transgene developed normally, whereas those expressing the A152T-tau transgene developed spinal curvature defects by 3 dpf (Supplementary Fig. 3B). To definitively address whether the WT-tau and A152T-tau transgenes were expressed at similar levels, we measured mRNA levels by quantitative RT-PCR on clutches from WT-tau and A152T-tau fish at 24 hpf (pre-phenotype) and correlated this with the distribution of morphological phenotypes present in the same clutches at 3 dpf (Fig. 2G, Supplementary Fig. 4A–C). Notwithstanding the variability found in the levels of expression of both Gal4 and Dendra-tau in different clutches, high levels of Dendra-tau mRNA expression were observed in WT-tau fish that always displayed a normal phenotype, whereas lower levels of Dendra-tau mRNA expression at 24 hpf (pre-phenotype) in A152T-tau fish resulted in subsequent phenotypic abnormalities at 3 dpf (Supplementary Fig. 4C). This was further verified by identifying clutches of fish having equivalent levels of Dendra-tau mRNA at 24 hpf [clutches 2 and 5, Fig. 2G(i)] and, as previously observed, only those expressing the A152T-tau transgene developed morphological abnormalities [Fig. 2G(ii)]. When mRNA expression levels were measured in individual fish, we observed no difference in transgene expression at 24 hpf. Similarly, at 3 dpf, no differences in mRNA expression levels were observed between fish expressing WT-tau and fish expressing A152T-tau with moderate and severe phenotypes. Interestingly, we observed significantly lower expression levels in morphologically normal A152T-tau fish and this is likely to account for the lack of defects observed in these individuals (Fig. 2H). Together, these findings confirmed that the defects observed in A152T-tau fish were not a consequence of higher mRNA expression levels but are specific to the mutant (A152T) form of the tau protein.

### A152T-tau zebrafish show increased tau-associated pathology

**Figure 3:**
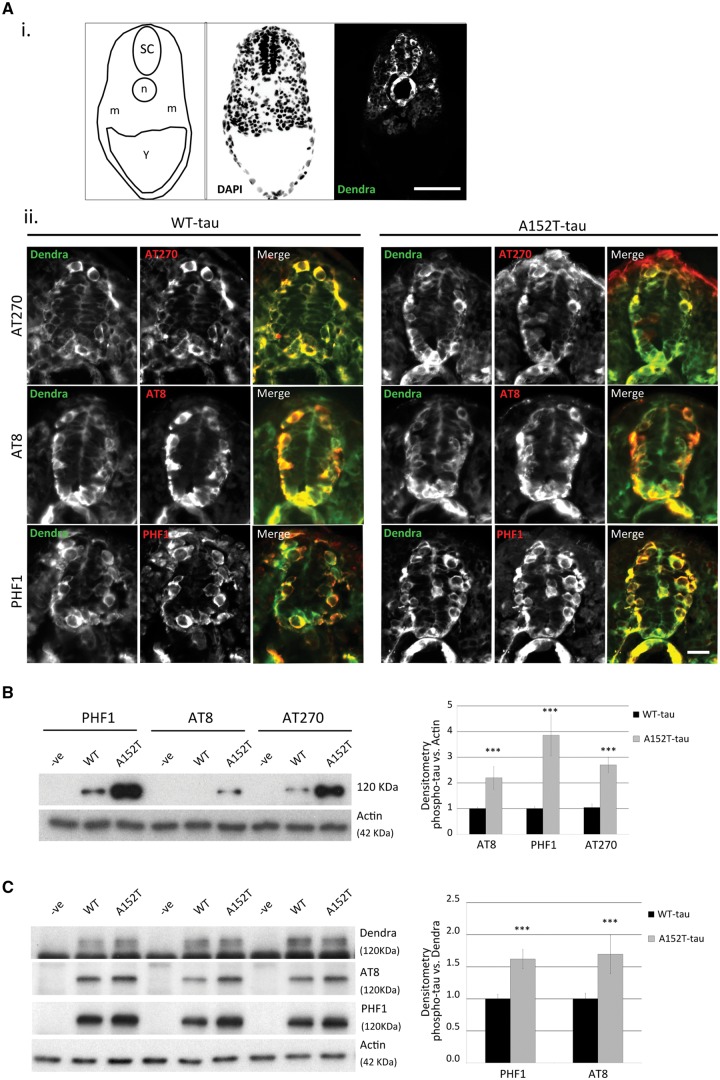
**Phosphorylation state of tau in Dendra-tau transgenic zebrafish.** Pathological hyperphosphorylation and conformational changes are hallmarks of tauopathies. (**A**) Both WT- and A152T-tau expressing larvae showed positive immunostaining for the hyperphosphorylation markers AT270 (residue Thr181), AT8 (residues Ser202/Thr205) and PHF1 (residues Ser396/Ser404) in cryosections, from 24 hpf onwards. [**A**(**i**)] Schematic overview, DAPI and Dendra images of transverse sections through the spinal cord used for phosphorylated tau detection at 24 hpf (SC = spinal cord, n* = *notochord, m = muscle block and Y = yolk sac). Scale bar = 50 μm. [**A**(**ii**)] Fluorescent images of Dendra-tau (green) and phospho-tau antibodies (red) show positive single neurons stained for AT270, AT8 and PHF1 within the spinal cord in both WT-tau and A152T-tau fish at 24 hpf. Scale bar = 10 μm. See Supplementary Fig. 5. (**B**) Western blot for phosphorylation markers AT270, AT8 and PHF1 in whole fish lysates at 3 dpf. The levels of phospho-tau were significantly increased in A152T-tau fish compared to WT-tau fish relative to the loading control, actin (mean ± SEM of 10 independent clutches; two-tailed *t*-test, ^***^P < 0.001 versus WT-tau). (**C**) The higher phosphorylation levels were also observed in A152T-tau fish relative to total tau (Dendra-tau) levels (mean ± SEM, *n = *24 fish/group of 10 independent clutches for PHF1 and eight independent clutches for AT8; two-tailed *t*-test, ^***^P < 0.001 versus WT-tau).

**Figure 4:**
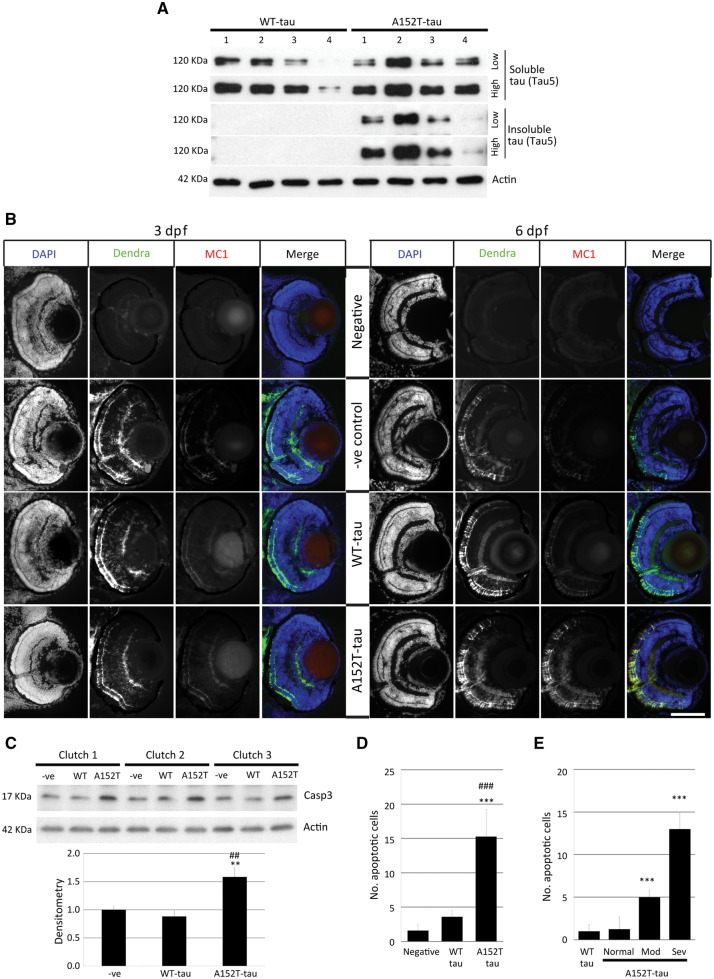
**Tau aggregation and cell death in Dendra-tau transgenic zebrafish**. (**A**) Levels of sarkosyl-soluble and insoluble tau reflect accumulation of the insoluble form only in A152T-tau fish at 6 dpf. The level of total tau was analysed by immunoblotting using Tau5 antibody (four independent clutches for WT-tau and A152T-tau). (**B**) Antibody staining for the conformational marker MC1 in cryosections across the eye of WT-tau and A152T-tau fish. No staining was observed in either WT-tau or A152T-tau fish at 3 dpf (*left*), whereas only A152T-tau presented positive staining for conformational changes at 6 dpf (*right*). Scale bar = 100 μm. (**C**) Western blot for active Caspase 3 (Casp3) (quantified below), indicative of increased cell death in fish expressing the A152T variant (mean ± SEM of nine independent clutches*;* Student-Newman-Keuls one-way ANOVA, *P < 0.01 versus negative, ^##^*P < *0.01 versus WT-tau). (**D**) The increased cell death in A152T-tau fish was confirmed by quantification of TUNEL labelling on transverse sections (mean ± SD; *n = *5 fish, from a minimum of five sections; Student-Newman-Keuls one-way ANOVA, ^***^P < 0.001 versus negative; ^###^*P < *0.001 versus WT-tau). See also Supplementary Fig. 4. (**E**) Morphologically abnormal A152T-tau fish showed increased cell death (quantification of TUNEL-positive nuclei) compared to morphologically normal A152T- or WT-tau fish (mean ± SD; Student-Newman-Keuls one-way ANOVA, ^**^P < 0.01 and ^***^P < 0.001 versus WT-tau). Representative images in Supplementary Fig. 7.

We next investigated the two transgenic lines for evidence of tau-associated pathology. Pathological hyperphosphorylation of tau is a hallmark of tauopathies (Iqbal *et al.*, 2010). Both transgenic WT-tau and A152T-tau fish showed positive staining for the hyperphosphorylation markers AT270 (residue Thr181), AT8 (residues Ser202/Thr205) and PHF1 (residues Ser396/Ser404) by whole-mount immunostaining (Fig. 3A and Supplementary Fig. 5). By western blotting, we observed that phosphorylation at multiple sites was increased in the A152T-tau fish relative to total protein (actin) levels and total tau (Dendra) levels (Fig. 3B and C). To determine whether transgene expression resulted in the formation of tau aggregates, we performed analysis of soluble and sarkosyl-insoluble tau. An abundance of sarkosyl insoluble tau was observed only in samples from A152T-tau fish (Fig. 4A). In addition, positive MC1 antibody staining, a marker of tau conformational change, was detected only in A152T-tau samples at 6 dpf and not in WT-tau transgenic fish (Fig. 4B). We also observed thioflavin-S staining, a well-established staining technique for neurofibrillary tangles (Guntern *et al.*, 1992), which are a characteristic of tau pathology, to be more abundant in the A152T-tau fish (Supplementary Fig. 6). Likewise, the levels of activated caspase 3, a marker for apoptosis (Fig. 4C), and number of apoptotic cells were increased in the A152T-tau zebrafish, and correlated with the morphological abnormalities (Fig. 4D and E and Supplementary Fig. 7). Together, these results demonstrate that A152T-tau expression is associated with greater tau-associated pathology and neurodegeneration.

### Delayed clearance of A152T-tau is associated with defects in proteasome function but not autophagy

**Figure 5:**
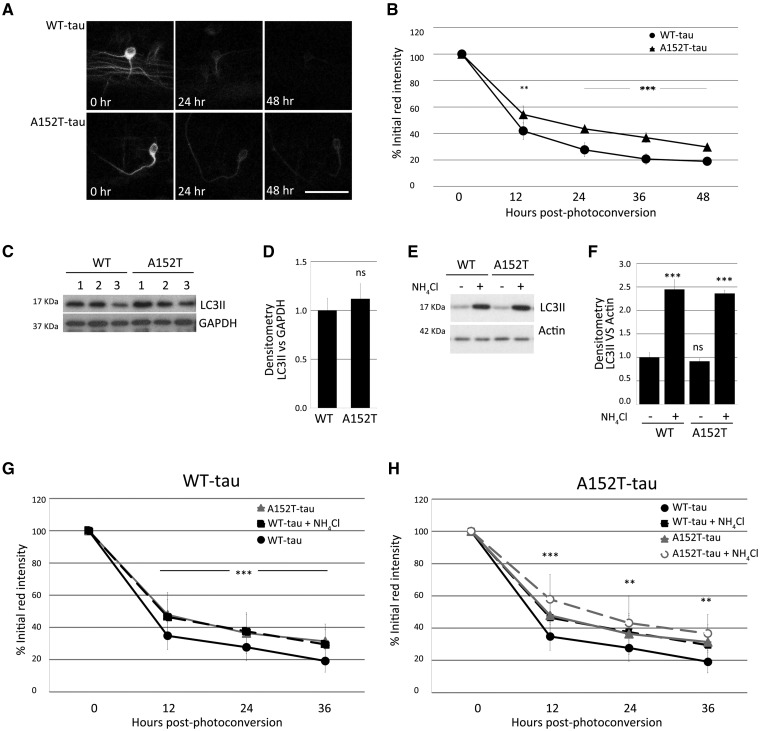
**Tau clearance *in vivo* and autophagy function.** Clearance kinetics of photoconverted Dendra-tau measured in neurons of WT-tau and AT152T-tau fish. Measurement of the intensity of the red Dendra-tau signal over time reflects the clearance or degradation of tau protein. (**A**) Representative images of photoconverted red Dendra-tau signal comparing a single neuron from WT-tau and A152T-tau fish at three different timepoints: immediately after photoconversion (0 h), 24 h and 48 h after photoconversion. (**B**) Quantification of red Dendra-tau intensity in photoconverted neurons in the spinal cord of WT-tau and A152T-Tau transgenic fish (representative images shown in Supplementary Fig. 8C). The percentage of residual photoconverted red Dendra-tau was measured over 48 h measured at 12-h intervals. Dendra-tagged A152T-tau clears at a significantly lower rate than WT-tau. (*n = *30/group shown as mean ± SD; Student-Newman-Keuls one-way ANOVA, ^**^P < 0.01 and ^***^P < 0.001 versus WT-tau). (**C–F**) Western blots for LC3-II, a well-characterized marker of autophagosome number, demonstrate that there are no differences in the levels of this protein between WT-tau and A152T-tau fish either at 24 hpf (pre-phenotype; **C** and **D**) or 72 hpf (post-phenotype; **E** and **F**). (**E** and **F**) Measurements of LC3-II levels in the presence or absence of ammonium chloride provides a method for measuring autophagic flux. No differences were observed between the two transgenic lines at 3 dpf, suggesting that autophagy functions normally in both WT-tau and A152T-tau fish (graph represents mean ± SD of four independent clutches per group for **E** and **F** and three for **C** and **D**; two-tailed *t*-test). (**G** and **H**) Clearance kinetics of Dendra-tau was measured in the presence or absence of ammonium chloride. Treatment with ammonium chloride blocks autophagic flux and delays the clearance of both WT-tau and A152T-tau to the same extent, indicating that flux occurs at the same rate in these two lines (mean ± SD, *n = *62 neurons/group; Student-Newman-Keuls one-way ANOVA, ^**^P < 0.01 and ^***^P < 0.001 versus untreated group). Note in **G** and **H**, the ‘WT-tau + NH_4_Cl’ (denoted by black squares and black dashed line) overlaps with the ‘A152T-tau’ line (denoted by grey triangles and grey solid line). The graphs in **G** and **H** are presented with a different line in the foreground and background to aid interpretation.

To study the influence of the A152T variant on tau turnover rate, we expressed Dendra-tau under the control of mosaic ubiquitous EIF1α::Gal4VP16 driver, as the mosaic expression enables analyses in single cells. Quantification of Dendra, a green-to-red photoconvertible fluorescent protein fused to tau, enabled assessment of tau clearance kinetics in individual spinal cord motor neurons *in vivo.* Mosaic fish were imaged before and after photoconversion then at defined intervals thereafter, to assess clearance of the red, photoconverted, fluorescently-tagged tau protein (Supplementary Fig. 8). Interestingly, A152T-tau showed significantly slower clearance kinetics than the WT-tau *in vivo* (Fig. 5A and B). As tau protein is known to be a substrate for both the proteasome and for autophagic degradation (Berger *et al.*, 2006), the slower clearance of A152T-tau may be the result of defects in either of these processes. However, autophagy appeared to be normal in WT-tau and A152T-tau fish as we observed no difference in levels of LC3-II, a well-characterized marker of autophagosome number, either in the presence or absence of ammonium chloride (Fig. 5C–F). Furthermore, when clearance experiments were performed in the presence of ammonium chloride, the rate of clearance was slowed to the same extent in both WT-tau and A152T-tau fish, suggesting that autophagy is functioning normally (Fig. 5G and H).

**Figure 6:**
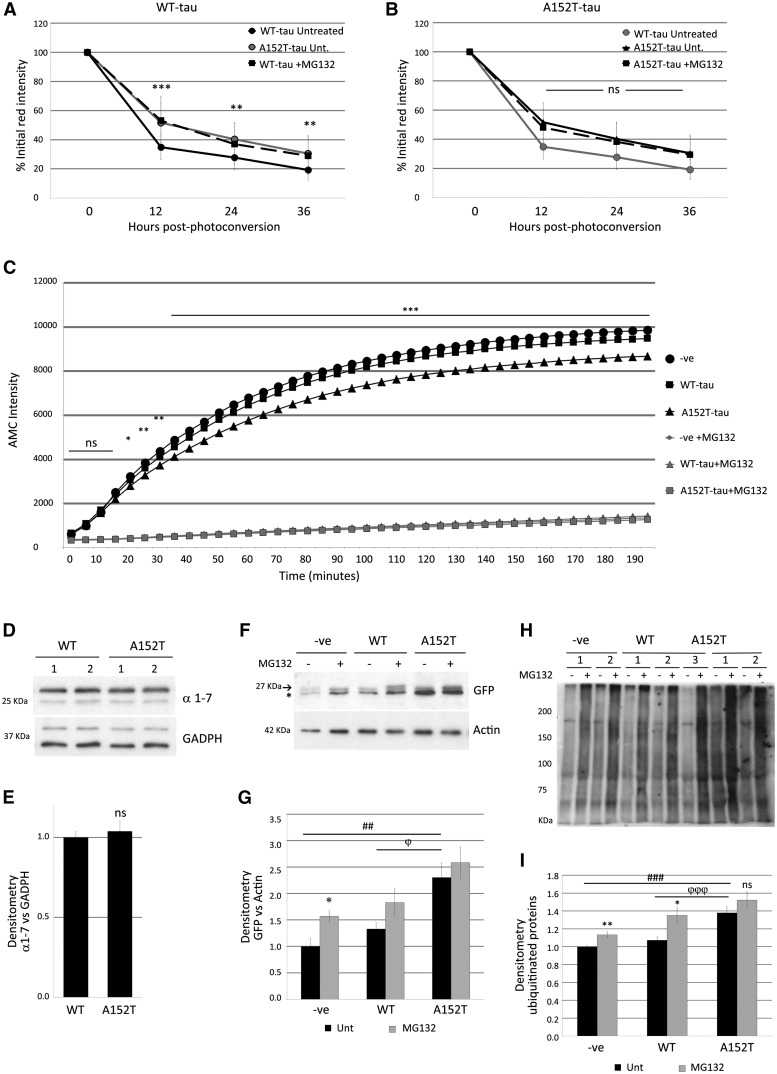
**Analysis of proteasome function in Dendra-tau transgenic zebrafish.** (**A** and **B**) Clearance kinetics of Dendra-tau in the presence or absence of MG132. Treatment with MG132 blocks proteasome function and delays the clearance of WT-tau (**A**) but had no effect on the clearance of A152T-tau (**B**) (*n = *65 neurons/group; mean ± SD; Student-Newman-Keuls one-way ANOVA, ^**^P < 0.01 and ^***^P < 0.001 versus untreated group). Note in **A**, the ‘WT-tau + MG132’ (denoted by black squares and black dashed line) overlaps with the ‘A152T-tau untreated’ line (denoted by grey circles and grey solid line). In **B**, the ‘A152T-tau untreated’ line (denoted by the black triangles and solid black line) overlaps with the ‘A152T-tau + MG132’ line (denoted by black squares and dashed black line). (**C**) Proteasome activity in lysates of WT-tau and A152T-tau fish was measured using the synthetic Suc-LLVY-AMC substrate. A152T-tau fish showed significantly reduced chymotrypsin-like activity compared to WT-tau fish (*n = *20 fish from six independent clutches per group in triplicate; mean ± SEM; two-tailed *t*-test: *P < 0.05; ^**^P < 0.01 and ^***^P < 0.001 versus WT-tau). When treated with MG132, proteasome function is blocked in all genotypes [i.e. non-transgenic fish (−ve), WT-tau and A152T-tau], hence the grey lines overlap. (**D** and **E**) Western blots for the α1–7 proteasome subunits showed no difference between WT-tau and A152T-tau fish indicating that the proteasome is structurally normal and equally abundant in both groups (lysates are the same as used for **C**; all bands are specific and upper band was used for quantification; mean ± SEM *n = *6/group; two-tailed *t*-test). (**F** and **G**) Transient expression of UbG76V-GFP (Dantuma *et al.*, 2000) was used to measure proteasome function *in vivo.* In non-transgenic and WT-tau transgenic fish, the GFP signal is detected at a low level under basal conditions but is increased in the presence of MG132. However, in A152T-tau fish the basal level of GFP is higher than in non-transgenic and WT-tau fish and treatment with MG132 fails to increase the detected levels of GFP (specific band found at 27 kDa, asterisk shows unspecific band in **F**; mean ± SEM of three clutches per group, *n = *20 fish per clutch in duplicate; two-tailed *t*-test, *P < 0.05 versus untreated group, ^Φ^*P < *0.05 versus untreated WT-tau group, ^##^*P < *0.001 versus untreated negative siblings). (**H** and **I**) Higher levels of endogenous ubiquitinated proteins were detected in A152T-tau fish compared to WT-tau fish (minimum two independent clutches per group, each analysed in triplicate, mean ± SEM; two-tailed *t*-test, *P < 0.05 and ^**^P < 0.01 versus untreated group, ^ΦΦΦ^*P < *0.001 versus untreated WT-tau and ^###^*P < *0.001 versus untreated negative siblings).

However, treatment with the proteasome inhibitor MG132 resulted in different effects on the clearance kinetics of WT-tau and A152T-tau fish. While MG132 treatment slowed the clearance of WT-tau (Fig. 6A), it had no effect on the clearance of A152T-tau, suggesting that proteasome activity may be suppressed in fish expressing the A152T variant (Fig. 6B). To investigate this further, we measured proteasome activity using a synthetic proteasome substrate and found significantly reduced activity in the A152T-tau fish lysates compared to those from WT-tau fish (Fig. 6C). This is likely a result of reduced proteasome function, as a structural component of the proteasome was equally abundant in both transgenic lines (Fig. 6D and E). To investigate proteasome activity *in vivo*, we used transient expression of UbG76V-GFP (Dantuma *et al.*, 2000) in fish with ubiquitous expression of WT-tau or A152T-tau. The presence of the ubiquitin sequence linked to GFP targets the fluorescent protein for rapid degradation by the proteasome under normal conditions, however, when proteasome function is perturbed then GFP is detected. In non-transgenic fish, the GFP signal is barely detected under basal conditions but was evident in the presence of MG132, with similar effects observed in WT-tau fish. However, in A152T-tau fish, the basal level of GFP was higher than that of non-transgenic and WT-tau fish (Fig. 6F and G) suggesting that the proteasome is impaired in these fish. In addition, levels of endogenous ubiquitinated proteins were higher in A152T-tau fish compared to WT-tau fish, and treatment with MG132 failed to increase the levels of ubiquitin-conjugated proteins further (Fig. 6H and I), suggesting that there was accumulation of proteins which are predominantly substrates of the proteasome.

### Delayed clearance of A152T-tau is associated with pathology and can be ameliorated by enhancing autophagy

**Figure 7:**
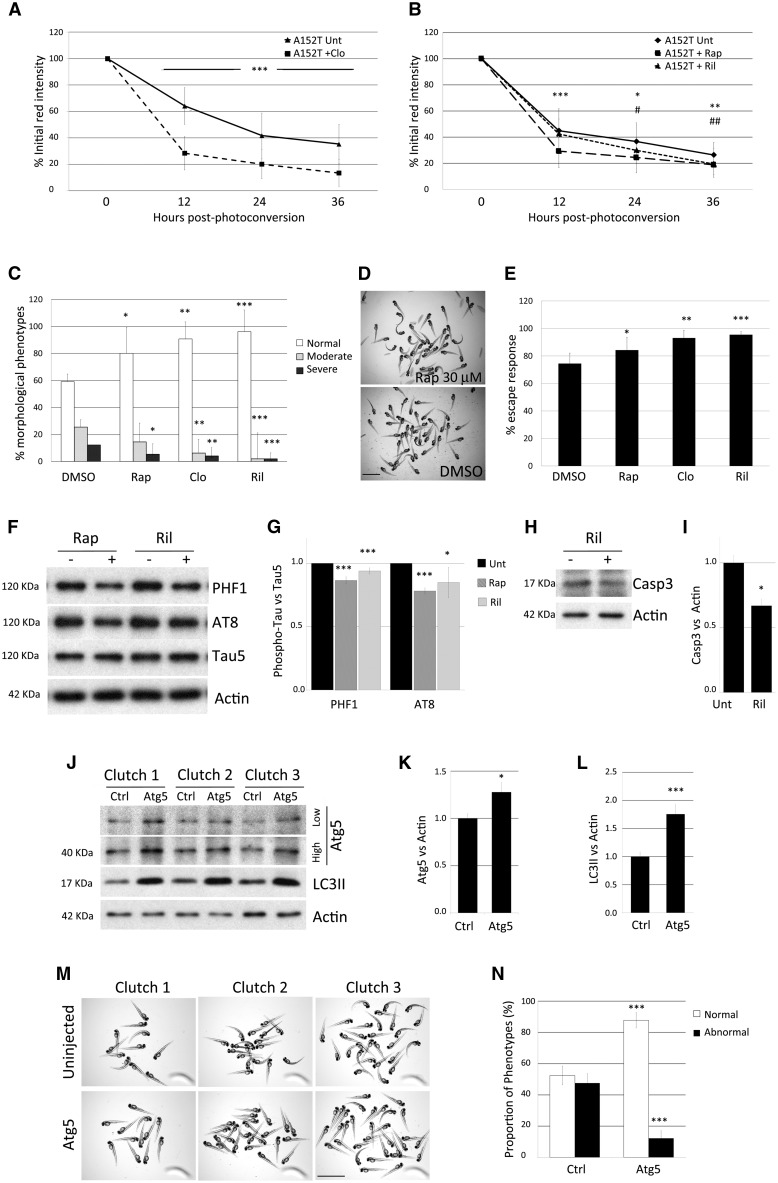
**Modulation of A152T-tau clearance and pathology by upregulation of autophagy.** (**A** and **B**) Treatment of A152T-tau fish with the autophagy inducers clonidine (**A**), rapamycin or rilmenidine (**B**), accelerated clearance kinetics of A152T-tau (mean ± SD of *n* ≥ 40 neurons/group; Student-Newman-Keuls one-way ANOVA, *^/#^*P < *0.05; ^**^^/##^*P < *0.01 ^***^P < 0.001 versus untreated). (**C**) Treatment with rapamycin (rap), clonidine (clo) or rilmenidine (ril) also ameliorated morphological defects in A152T-tau transgenic fish (*n = *6 independent experiments, 20 fish/group, mean ± SD; Student-Newman-Keuls one-way ANOVA, *P < 0.05; ^**^P < 0.01 and ^***^P < 0.001 versus DMSO). (**D**) Representative image showing rapamycin treatment reduced the proportion of abnormal A152T-tau fish. Scale bar = 3.5 mm. (**E**) Quantification of the escape response measured in individual A152T-tau fish 3 dpf, treated with either DMSO, rapamycin, clonidine or rilmenidine. (*n = *5 independent experiments in triplicate, 15/group shown as mean ± SD; two-tailed *t*-test: *P < 0.05, ^**^P < 0.01 and ^***^P < 0.001 versus DMSO). The treatment with autophagy upregulators improved the escape response deficit in A152T-tau fish. (**F** and **G**) Induction of autophagy by the addition of rapamycin or rilmenidine to A152T-tau fish also reduced levels of phosphorylated tau at residues Ser202/Thr205 (AT8) and Ser396/Ser404 (PHF1) relative to total tau levels (*n = *3 independent experiments in duplicate, 10/group shown as mean ± SEM; two-tailed *t*-test: *P < 0.05 and ^***^P < 0.001 versus DMSO). (**H** and **I**) Treatment with autophagy upregulators results in less cell death. Rilmenidine treatment reduced the levels of active caspase-3 (*n = *4 independent experiments in triplicate, 10/group shown as mean ± SEM; two-tailed *t*-test: *P < 0.05 versus DMSO). (**J–N**) Injection of an expression vector encoding zebrafish *atg5* into A152T-tau fish embryos resulted in over-expression of Atg5 protein at 2 dpf (**J** and **K**) (high and low exposure of the same blot presented; mean ± SD, *n = *6 independent clutches; two-tailed *t*-test, *P < 0.05 versus control). (**J** and **L**) The increase in Atg5 protein correlated with increase in LC3II, a well-characterized reporter for autophagosome number, demonstrating that autophagy was upregulated in Atg5-injected fish (mean ± SEM, *n = *8 independent clutches; two-tailed *t*-test, ^***^P < 0.001 versus control). (**M** and **N**) A consistent reduction in the number of offspring with morphological defects was observed in Atg5-injected fish compared to control (uninjected) siblings. The percentage of normal fish changes from 52.43% ± 6.01 to 87.79% ± 4.87 after Atg5 injection and consequently, the proportion of abnormal fish diminishes from 47.57% ± 6.01 to 12.21% ± 4.87 (mean ± SEM of seven independent clutches; two-tailed *t*-test, ^***^P < 0.001 versus control).

As (macro)autophagy induction can enhance the clearance of tau (Berger *et al.*, 2006; Moreau *et al.*, 2014) and autophagic flux was normal in our transgenic zebrafish (Fig. 5C–H), we tested the effects of clonidine, rilmenidine and rapamycin, known autophagy inducers (Williams *et al.*, 2008), on the clearance of A152T-tau. This pharmacological autophagy upregulation enhanced the clearance of A152T-tau (Fig. 7A and B), rescued the morphological abnormalities of the fish (Fig. 7C and D) and ameliorated the motility defects (Fig. 7E). Furthermore, autophagy upregulation also resulted in a reduction in hyperphosphorylated tau (Fig. 7F and G) and in lower activated caspase3 levels in treated versus untreated A152T-tau fish (Fig. 7H and I).

To further validate that upregulation of autophagy ameliorated defects in A152T-tau fish, we used a genetic approach using transient over-expression of the key autophagy gene *atg5.* Injection of an expression vector encoding zebrafish Atg5 into A152T-tau fish at the one cell stage resulted in over-expression of Atg5 protein at 2 dpf (Fig. 7J and K). The increase in Atg5 protein correlated with increase in LC3-II, a well-characterized reporter for autophagosome number, demonstrating that autophagy was upregulated in Atg5-injected fish (Fig. 7J and L). Phenotypic analysis of Atg5-injected and control (uninjected) siblings demonstrated that increased autophagy was associated with a reduction in the number of offspring with morphological defects (Fig. 7M and N).

## Discussion

Confirmation of genetic associations and functional exploration of the biology of rare variants will be become increasingly important as the scientific community seeks to utilize genetic data. Here, we have focused on the p.A152T *MAPT* variant. After the initial study that reported significant risks for p.A152T carriers to develop Alzheimer’s disease and FTD spectrum disorders (Coppola *et al.*, 2012), Labbe *et al.* (2015) reported significant associations with cohorts with clinically-diagnosed or pathologically-confirmed dementia with Lewy bodies. However, Pastor *et al.* (2016) did not show a significant association between p.A152T and Alzheimer’s disease, and the association with FTD was of borderline significance (OR = 2.03; 95% CI = 0.95–4.34 *P = *0.063) (Pastor *et al.*, 2016).

Using a large multinational consortium, we attempted to clarify the situation with regard to FTD spectrum and other clinical syndromes known to be caused by FTLD-tau. In the present study, we find significant risk associations in our independent FTD and PSP-S cohorts with the p.A152T variant. This replicates the effect for FTD we reported initially (Coppola *et al.*, 2012) and is consistent with the trend observed by Pastor *et al.* (2016). Thus, we feel that this constitutes good genetic support for p.A152T as a tauopathy risk variant. Previous neuropathologic analysis of autopsies of individual patients carrying the p.A152T MAPT variant have shown different types of abnormal tau accumulation, some fulfilling criteria for corticobasal degeneration, PSP (Kara *et al.*, 2012), or pallidonigroluysian atrophy (Graff-Radford *et al.*, 2013), while others were considered to be unclassifiable tauopathies (Kovacs *et al.*, 2011). It remains unclear whether and how the *MAPT* variant relates to diseases without predominant tau pathology, although we acknowledge that larger samples may be able to reveal associations with other conditions, like Parkinson’s disease and Lewy body dementia.

Previous studies have suggested that p.A152T tau may diminish tau binding to microtubules, while increasing tau oligomer formation (Coppola *et al.*, 2012) and cause increased tau phosphorylation in induced pluripotent stem cell-derived neurons, among other phenotypes. To test p.A152T pathogenicity *in vivo*, we developed zebrafish models to compare WT-tau to A152T-tau alleles. The A152T-tau fish have increased tau phosphorylation, insoluble tau and neurofibrillary tangle formation associated with increased caspase staining (apoptosis) and neurodegeneration. Similarly, the A152T-tau fish had overt morphological and behavioural defects compared to the WT-tau counterparts. Our data are compatible with the more severe phenotypes seen in mouse models expressing A152T compared to wild-type tau (Decker *et al.*, 2016; Maeda *et al.*, 2016).

We characterized the expression levels of the two transgenes (WT-tau and A152T-tau) and have robustly demonstrated that the pathology observed in fish expressing the A152T-tau variant is not a consequence of higher mRNA expression. However, we found protein levels of A152T-tau to be increased at the times when the pathological phenotypes manifest. Using a novel, *in vivo* protein clearance assay, we found that A152T-tau had slower clearance kinetics compared to WT-tau *in vivo*, and this could be attributed to impaired proteasome function. These effects of a disease-associated tau variant showing greater inhibition on the proteasome activity than wild-type tau are likely mediated via the same processes described recently for the P301L autosomal dominant tau mutation (Myeku *et al.*, 2016) and this inhibition of proteasome activity may provide a mechanism to account for the higher protein/mRNA ratios observed in A152T mice compared to those expressing WT-tau (Maeda *et al.*, 2016).

This proteasome impairment may explain an important conundrum; namely why the A152T variant has been associated with TDP-43 and Lewy body/alpha-synuclein pathology in different isolated cases (Kara *et al.*, 2012). While these associations may be coincidence, the possibility that A152T tau may be able to provoke the accumulation of other intracytoplasmic proteins needs to be considered. This is especially relevant given the association data of Labbe *et al.* (2015) implicating A152T in alpha-synucleinopathies, and the multitude of papers showing associations between the *MAPT*/tau haplotype and Parkinson’s disease risk (Desikan *et al.*, 2015). All of these studies raise the question of how a tau variant may provoke the accumulation of proteins besides tau.

We show that A152T-tau impairs proteasome activity, and this causes the accumulation of proteasome substrates (not only tau itself). Proteasome substrates also include alpha-synuclein (Webb *et al.*, 2003) and TDP-43 (Huang *et al.*, 2014). So, we propose that a primary consequence of A152T-tau is to impair to activity of the proteasome, and cause the accumulation of various proteasome substrates. As this is a variant that acts via incomplete penetrance and likely requires other genetic and environmental factors to enable disease in humans, we hypothesize that, depending on the genetic background effects on the biologies and metabolism of other proteins like alpha-synuclein or TDP-43, these could accumulate more rapidly than tau in some cases with the A152T variant. A similar mechanism may be relevant even to the situation of the tau haplotype and alpha-synucleinopathies, as our data suggest that even overexpression of wild-type tau in zebrafish (which was not associated with any gross phenotypes) led to some proteasome impairment, although this was less marked than that seen with the A152T variant. Thus, our mechanistic data allow an understanding of a mystery with regard to the influence of tau on diseases other than pure tauopathies.

As autophagy was not obviously perturbed in our model, we tested whether enhancing tau clearance by upregulating autophagy would be able to reduce the severity of the A152T-tau phenotypes. Using both pharmacological and genetic autophagy enhancers, we could demonstrate phenotypic amelioration in the A152T-tau fish as well as changes in hyperphosphorylation and cell death, suggesting that this therapeutic strategy deserves consideration. For therapeutic efficacy in humans, one may not require constitutive autophagy upregulation, as pulsatile treatment may be effective (Ravikumar *et al.*, 2004; Rose *et al.*, 2010). Importantly, this strategy reduces the load of the toxic protein for the relevant cells, thus targets the primary disease-initiating factor.

In conclusion, we have performed a large genetic association study that now replicates and confirms the increased risk for FTD and PSP-S conferred by the p.A152T tau allele. We have supported these data with this rare variant with animal modelling in zebrafish that demonstrate clear increased pathogenicity of this risk allele. Furthermore, our data suggest that autophagy induction may be a suitable therapeutic strategy for these tauopathies.

## Supplementary Material

Supplementary Data

## Abbreviations

d/hpf: days/hours post-fertilization
FTD: frontotemporal dementia
FTLD: frontotemporal lobar degeneration
PSP-S: progressive supranuclear palsy syndrome
TUNEL: terminal deoxynucleotidyl transferase dUTP nick end labelling
