# Corrigendum

**DOI:** 10.1093/brain/awx072

**Published:** 2017-03-25

Ana Lopez, Suzee E. Lee, Kevin Wojta, Eliana Marisa Ramos, Eric Klein, Jason Chen, Adam L. Boxer, Maria Luisa Gorno-Tempini, Daniel H. Geschwind, Lars Schlotawa, Nikolay V. Ogryzko, Eileen H. Bigio, Emily Rogalski, Sandra Weintraub, Marsel M. Mesulam, Angeleen Fleming, Giovanni Coppola, Bruce L. Miller, David C. Rubinsztein. A152T tau allele causes neurodegeneration that can be ameliorated in a zebrafish model by autophagy induction. Brain 2017; 140: 1128–1146; 10.1093/brain/awx005.

The authors apologize for an error in in the author list, which should read as follows:

Ana Lopez,^1,2,#^ Suzee E. Lee^3,#^ Kevin Wojta^4,#^ Eliana Marisa Ramos,^4^ Eric Klein,^4^ Jason Chen,^4^ Adam L. Boxer,^3^ Maria Luisa Gorno-Tempini,^3^ Daniel H. Geschwind,^4^ Lars Schlotowa,^1,2^ Nikolay V. Ogryzko,^5^ Eileen H. Bigio,^6^ Emily Rogalski,^6^ Sandra Weintraub,^6^ Marsel M. Mesulam,^6^ Tauopathy Genetics Consortium,^† ^Angeleen Fleming,^1,2,^* Giovanni Coppola,^4,^* Bruce L. Miller^3,^* and David C. Rubinsztein^1,^*

^#,^*These authors contributed equally to this work.

^†^See Appendix 1.

The Appendix was mistakenly omitted from the manuscript, and should read as follows:

**Appendix 1**

**Tauopathy Genetics Consortium**

Federica Agosta, Antonella Alberici, Gülsen Babacan-Yildiz, David A. Bennett, Eileen H. Bigio, Kaya Bilguvar, Barbara Borroni, Adam L. Boxer, Ahmet O. Caglayan, Onofre Combarros, Giancarlo Comi, Giovanni Coppola, Etty P. Cortés, Isidre Ferrer, Şermin Genç, Daniel H. Geschwind, Murat Gunel, Karen H. Gylys, Begoña Indakoetxea, Clementine E. Karageorgiou, Anna Karydas, Ulkan Kilic, Suzee Lee, Adolfo Lopez De Munain, Giuseppe Magnani, Robert W. Mahley, Filippo Martinelli Boneschi, Jacqueline Martinez, Salvatore Mazzeo, Marsel M. Mesulam, Bruce L. Miller, Fermin Moreno, Alessandro Padovani, John Papatriantafyllou, Ekaterina Rogaeva, Emily Rogalski, Pascual Sanchez-Juan, Roberto Santangelo, Gary W. Small, Rawan Tarawneh, Maria Carmela Tartaglia, Jean Paul G. Vonsattel, Sandra Weintraub, Gorsev Yener.

See Supplementary material for affiliations.

