# Supplementary material for: A152T tau allele causes neurodegeneration that can be ameliorated in a zebrafish model by autophagy induction
Source: Brain. 2017 Feb 9;140(4):1128–46. doi: 10.1093/brain/awx005 (PMC5382950; doi:10.1093/brain/awx005)
Supplement: Supplementary Data [file awx005_supp.pdf]

Supplementary Appendix

Tauopathy Genetics Consortium

Federica Agosta[1], Antonella Alberici[2], Gülsen Babacan-Yildiz[3], David A. Bennett[4], Eileen H. Bigio[5], Kaya Bilguvar[6], Barbara Borroni[2], Adam L. Boxer[7], Ahmet O. Caglayan[8,9], Onofre Combarros[10,11], Giancarlo Comi[12], Giovanni Coppola[13], Etty P. Cortés[14], Isidre Ferrer[15], Şermin Genç[16], Daniel H. Geschwind[13], Murat Gunel[8], Karen H. Gylys[17], Begoña Indakoetxea[10,18], Clementine E. Karageorgiou[19], Anna Karydas[7], Ulkan Kilic[20], Suzee Lee[7], Adolfo Lopez De Munain[10,18], Giuseppe Magnani[12], Robert W. Mahley[21], Filippo Martinelli Boneschi[22], Jacqueline Martinez[13], Salvatore Mazzeo[12], Marsel M. Mesulam[5], Bruce L. Miller[7], Fermin Moreno[10,18,23], Alessandro Padovani[2], John Papatriantafyllou[24], Ekaterina Rogaeva[25], Emily Rogalski[5], Pascual Sanchez-Juan[10,11], Roberto Santangelo[12], Gary W. Small[13], Rawan Tarawneh[26], Maria Carmela Tartaglia[25], Jean Paul G. Vonsattel[14], Sandra Weintraub[5], Gorsev Yener[16,27]

Affiliations:

[1] Neuroimaging Research Unit, Institute of Experimental Neurology, Division of Neuroscience, San Raffaele Scientific Institute, Milan, Italy

[2] Neurology Unit, Department of Clinical and Experimental Sciences, University of Brescia, Brescia, Italy

[3] Istanbul Bezmialem Vakif University Medical School, Adnan Menderes Bulvarı, Vatan Caddesi 34093 Istanbul, Turkey

[4] Rush Alzheimer's Disease Center, Rush University Medical Center, Chicago, IL, USA

[5] Cognitive Neurology and Alzheimer’s Disease Center, Northwestern University Feinberg School of Medicine, Chicago, IL 60611, USA

[6] Yale Center for Genome Analysis, Yale School of Medicine, West Haven, CT, USA

[7] University of California San Francisco Memory and Aging Center, San Francisco, CA, USA

[8] Departments of Neurosurgery, Neurobiology and Genetics, School of Medicine, Yale University, New Haven, CT, USA

[9] Department of Medical Genetics, School of Medicine, Istanbul Bilim University, 34394, Istanbul, Turkey

[10] Centro de Investigación Biomédica en Red de Enfermedades Neurodegenerativas, CIBERNED, Instituto de Salud Carlos III, Madrid, Spain

[11] 'Marqués de Valdecilla' University Hospital, Institute for Research 'Marqués de Valdecilla' (IDIVAL), University of Cantabria, Cantabria, Spain

[12] Department of Neurology, Institute of Experimental Neurology, Division of Neuroscience, San Raffaele Scientific Institute, Vita-Salute San Raffaele University, Milan, Italy

[13] Semel Institute for Neuroscience and Human Behavior, David Geffen School of Medicine, University of California Los Angeles, Los Angeles, CA 90024, USA

[14] Department of Pathology and Cell Biology, Columbia University Medical Center, New York, NY, USA

[15] Institute of Neuropathology, Service of Pathologic Anatomy, Bellvitge University Hospital, carrer Feixa Llarga s/n, 08907 Hospitalet de Llobregat, Spain

[16] Dokuz Eylul University, International Biomedicine and Genome Institute, Izmir, Turkey

[17] School of Nursing, University of California Los Angeles, Los Angeles, CA, USA

[18] Neuroscience Area, Institute Biodonostia, San Sebastian, Gipuzkoa, Spain

[19] Neurological Institute of Athens, Greece

[20] Istanbul Medipol University, Faculty of Medicine, Department of Medical Biology, Istanbul, Turkey

[21] Gladstone Institute of Cardiovascular Disease; Departments of Pathology and Medicine, University of California, San Francisco, San Francisco, CA 94143, USA

[22] Laboratory of Genetics of Neurological Complex disorders, Institute of Experimental Neurology, Division of Neuroscience, San Raffaele Scientific Institute, Milan, Italy

[23] Department of Neurology, Hospital Universitario Donostia, San Sebastian, Gipuzkoa, Spain

[24] Neurology Dpt., 'G.Gennimatas' Hospital of Athens, Greece

[25] University of Toronto, Tanz Centre for Research in Neurodegenerative Diseases, 60 Leonard Street, Toronto, ON Canada, M5T 2S8

[26] Cleveland Clinic Lou Ruvo Center for Brain Health, Neurological Institute, Department of Neurology, Cleveland Clinic Main Campus, Cleveland, OH 44195, USA

[27] Dokuz Eylul University, Medical School, Department of Neurology, Izmir, Turkey

Funding: GCoppola, DHG, SL, ALB, BLM were supported by the Tau Consortium. ALB was supported by the Tau Consortium, U54NS092089 and R01AG038791. DAB was supported by P30AG10161, RF1AG15819, and R01AG17917. EHB, MMM, ER and SW were supported by P30AG13854; MMM, ER and SW by R01NS075075 and R01DC008552. FA,

FMB, GComi, GM, RS, SM were supported by the Italian Ministry of Health GR-2010-2303035. GWS was supported by the Fran and Ray Stark Foundation Fund for Alzheimer's Disease Research; PSJ was supported by a grant from FIS (PI12/02288) and JPND project DEMTEST (PI11/03028). MCT was supported by the Toronto General and Western Hospital Foundation.

Figure S1. Generation of transgenic Dendra-tau zebrafish:

(**A**) The responder construct comprises the gene encoding human wildtype or mutant A152T tau fused to the sequence encoding the photoactivatable protein Dendra, downstream of UAS. The transgene also contains EGFP driven by the cardiac myosin light chain (CMLC2) promoter in the reverse orientation. The Gal4 driver constructs contain the ubiquitous (EIF1α) or pan-neuronal (PanN) zebrafish promoters driving the expression of Gal4-VP16, which binds to the UAS on the responder construct. (**B**) To generate transgenic fish, the responder construct was injected together with the Tol2 mRNA (Bi) to facilitate random integration of the construct into the zebrafish genome resulting in mosaic founder embryos (Bii, scale bar represents 500 μm). Mosaic Dendra-tau positive larvae were identified at 3 d.p.f, raised and outcrossed with wild-type fish (Biii). Transgenic offspring were identified by green hearts i.e. expression from the CMLC2::EGFP reporter (Biv). Scale bar represents 1mm. (**C**) Dendra-tau expression patterns in the offspring of UAS::Dendra-tau fish crossed with EIF1α::Gal4VP16 and PanN:Gal4VP16 driver fish at 48 hours post-fertilisation (h.p.f). Dendra-tau driven by EIF1α::Gal4VP16 results in a ubiquitous but mosaic expression of Dendra-tau. Such crosses were used for clearance assays in Fig. 5A,B,G&H, Fig. 6A&B and Fig 7A&B. Ubiquitous transgene expression in offspring from crosses to beta-actin::Gal4VP16 driver fish were used for proteasome assays in Fig. 6F&G (Dendra-tau driven by beta-actin::Gal4VP16 not shown). All other experiments use Dendra-tau driven by PanN::Gal4VP16Scale bar represents 500 μm.

Figure S2. Cholinergic denervation in Dendra-tau fish

(**A**) Antibody staining for alpha-acetylated tubulin in 3 d.p.f. WT-tau and A152T-tau fish visualise abnormalities in branching of motor neurons (supporting main figure Fig.2B&C). Scale bar represents 100 μm. **(B)** Representative images showing the loss of cholinergic

(motor) neurons in the spinal cord (squared area) of A152T-tau fish compared to WT-tau by immunostaining with ChAT antibody (6 d.p.f.) (nc=notochord; m=muscle). Images iii and iv show high magnification regions of the spinal cord. Scale bar represents 50 µm. (**C**) Quantification of the number of ChAT-positive motor neurons across 20 sections of the spinal cord from the dorsal fin region (mean ± standard error, N=5 fish/group; two-tailed *t*-test, **P<0.01 *vs.* WT-tau). (**D**) Quantification of the escape response of mutant A152T-tau fish or Dendra-negative siblings at 3 d.p.f showing that defects are observed at 3 dp.f. in addition to those reported at 6 d.p.f. in Figure 2D (3 independent experiments in triplicate, N=20/group shown as mean ± standard error; ***P<0.001 *vs.* negative siblings by two-tailed *t*-test).

Figure S3. Dendra-intensity levels in Dendra-tau fish

(A) Quantification of the mean fluorescent intensity of Dendra in WT-tau and A152T-tau fish shows no significant differences at 24 h.p.f. (pre-phenotype) and fluorescent intensity does not correlate with the onset of morphological abnormalities in A152T-tau fish. **(B)** Images of WT-tau and A152T-tau fish sorted for similar initial Dendra fluorescent intensity at 24 h.p.f and followed over subsequent days. Only fish expressing A152T-tau developed abnormal phenotype. Scale bar represents 1 mm.

Figure S4. Analysis of expression levels in WT and A152T-tau zebrafish

(**A-C**) Further examples of quantification by Q-PCR of the expression levels of Dendra (black) and Gal4 (grey) in individual clutches from WT-tau and A152T-tau fish at 24 h.p.f. (pre-phenotype) (additional data to support that presented in Fig.2). The expression levels of Dendra and Gal4 vary between different clutches. However, results from experiments shown in A and B at 24 h.p.f show comparable variability in both groups (*Circles label samples presented in*

supplementary Figure 4C). (Ci) Quantification of the expression levels of Dendra and Gal4 by Q-PCR at 24 h.p.f (pre-phenotype) shows greater expression of Dendra in clutch 3 (WT-tau fish) than in clutches 4-6 (A152T-tau fish). (Cii) Phenotypic assessment of larvae at 3.d.p.f. from the same clutches analysed in (Ci) shows abnormal phenotypes in all clutches of A152T-tau fish regardless of the expression level of Dendra-tau (the different morphological phenotypes are as described in Figure 2, sev=severe, mod=moderate). (**D**) Correlation between Dendra and Tau5 antibodies for the detection of Dendra-tau by western blot.

Figure S5. Dendra-tau phosphorylation in transgenic zebrafish

Further data supporting main Fig.3. (**A**) Representative images of whole-mount immunostaining for the hyperphosphorylation marker AT8 at 24 and 48 h.p.f. in the areas represented in schematic overviews above. Pictures show Dendra signal (green) and positive AT8 staining (red) in both WT- and A152T-tau larvae from 24 h.p.f. Pictures at higher magnification demonstrate the presence of hyperphosphorylated tau in somas of individual motorneurons in the spinal cord (sc) as well as their axonal projections. At 48 h.p.f, the staining was greater in the axons. Scale bar represents 150 µm.

Figure S6. Dendra-tau aggregation in transgenic zebrafish

Further data supporting tau aggregation shown in main Fig.4. (**A**) Thioflavin-S staining of transverse sections through the eye and brain at the level of the optic chiasm. Positive staining was observed in both WT- and A152T-tau fish indicating tau aggregation and the presence of NFTs with increased accumulation in fish expressing the mutant A152T-tau (arrowheads). Upper panel, DAPI (nuclear) stained sections of eye and brain with selected regions stained with thioflavin-S presented at higher magnification below (i-iv show eye; i'-iv' brain). Scale bar represents 40 µm. Scale bar represents 20 µm.

Figure S7. Cell death in Dendra-tau zebrafish

Further data supporting main Fig.4. (**A**) Representative images of TUNEL labelling in brain sections showing consistent and reproducible differences in cell death between WT- and A152T-tau fish according to morphological phenotype (normal, moderate and severe) at 6 d.p.f. Apoptotic nuclei are highlighted by white arrowheads and quantification is shown in main Fig. 4E. **B**) TUNEL labelling on longitudinal sections of WT- and A152T-tau fish from 24 h.p.f. to 5 d.p.f. used to identify the time points at which cell death is occurring. The timecourse showed a larger number of apoptotic cells in mutant A152T-tau compared to WT-tau fish in all ages, most evident at 2 d.p.f. (**C**) TUNEL labelling in brain sections of WT- and A152T-tau fish at 2 d.p.f. representative of those used for quantification of the number of apoptotic cells presented in main Fig.4D (l=lens; r=retina and b=brain) . A-C scale bar represents 100 µm.

Figure S8. Dendra-photoconversion protocol

(**A**) Photoconversion of Dendra: Confocal images of green Dendra-tau and red Dendra-tau before and after photoconversion of spinal cord motor neurons of WT-tau fish. The circle indicates the area targeted for photoconversion with 405nm laser. Scale bar represents 50 µm. (**B**) Schematic diagram of the photoconversion protocol. On day 1, fish were screened at 24 h.p.f to identify those with mosaic Dendra-tau expression. On day 2, individual neurons in the spinal cord were exposed to 405nm light for 3 sec to photoconvert the green-Dendra into red-Dendra. Pictures of redDendra-tau signal were taken immediately after photoconversion (to obtain an image of the maximum red signal) and subsequently at 12, 24, 36 and 48 hr post-photoconversion. (**C**) Clearance of red-Dendra-tau signal: confocal images of photoconverted

neurons in WT-tau fish immediately after photoconversion and at 12, 24, 36 and 48 hours later. Intensity of the red signal was then analysed using FIJI software. A & C Scale bar represents 10 µm.

Supplementary Table 1. Demographic characteristics and *MAPT* A152T carrier frequencies in a series of patients and controls recruited worldwide across collaborating Centers.

Cohort	ALS	AD	CBS	FTD	MCI	PD	PSP-S	Control
**Allon**							243	
Carriers(%)							5 (2.06)	
Age at Onset							NA	
% Female							46.09	
% Caucasian							88.07	
**Gladstone (Turkish)**								2785
Carriers(%)								3 (0.11)
Age at Onset								-
% Female								NA
% Caucasian								100.00
**Italian**		54	51	167	16	1	40	29
Carriers(%)		0	0	0	0	0	0	0
Age at Onset		60.9	59.8	64.5	NA	58.0	NA	-
% Female		62.96	29.41	42.51	43.75	0	47.50	55.17
% Caucasian		100.00	100.00	100.00	100.00	100.00	100.00	100.00
**Northwestern**				172				
Carriers(%)				3 (1.74)				
Age at Onset				NA				
% Female				52.91				
% Caucasian				99.42				
**Rush**								204
Carriers(%)								0
Age at Onset								-
% Female								56.37
% Caucasian								97.55
**Small**		22		4	276			240
Carriers(%)		0		0	1 (0.36)			2 (0.83)
Age at Onset		NA		NA	NA			-
% Female		90.91		50.00	60.87			71.25
% Caucasian		86.36		100.00	73.91			87.92

Cohort	ALS	AD	CBS	FTD	MCI	PD	PSP-S	Control
**Spanish**		384	19	131		204	15	421
Carriers(%)		0	0	1 (0.76)		0	0	2 (0.48)
Age at Onset		63.3	NA	NA		63.8	NA	-
% Female		59.64	63.16	55.73		42.65	66.67	66.03
% Caucasian		100.00	100.00	100.00		100.00	100.00	100.00
**Toronto**				113				
Carriers(%)				0				
Age at Onset				NA				
% Female				26.55				
% Caucasian				43.36				
**Turkish**		53		13	1		11	366
Carriers(%)		0		0	0.00		1 (9.09)	3 (0.82)
Age at Onset		NA		NA	NA		NA	-
% Female		NA		NA	NA		NA	NA
% Caucasian		100.00		100.00	100.00		100.00	100.00
**UCSF**	24	350	62	257	158	1	86	247
Carriers(%)	0	3 (0.86)	0	3 (1.17)	1 (0.63)	0	2 (2.33)	0
Age at Onset	60.7	73.6	59.2	59.6	63.0	NA	62.5	-
% Female	33.33	53.14	59.68	43.58	48.10	0.00	50.00	57.89
% Caucasian	79.17	87.71	79.03	87.55	76.58	100.00	81.40	73.28
**Other**		64	1	56	11		40	59
Carriers(%)		2 (3.13)	1 (100.00)	0	0.00		0	0
Age at Onset		74.9	62.0	NA	81.00		NA	-
% Female		60.94	0.00	25.00	63.64		60.00	49.15
% Caucasian		35.94	100.00	98.21	81.82		2.50	42.37
**Overall**	24	927	133	913	462	206	435	4351
Carriers(%)	0	5 (0.54)	1 (0.75)	7 (0.77)	2 (0.43)	0	8 (1.84)	10 (0.23)
Age at Onset	60.7	69.50	59.70	63.0	63.40	63.7	62.5	-
% Female	33.33	54.80	48.12	43.04	55.84	42.23	47.82	17.28
% Caucasian	79.17	90.61	90.23	89.27	75.97	100.00	80.69	96.92

ALS , Amyotrophic Lateral Sclerosis; AD, Alzheimer's Disease; CBS, Corticobasal Syndrome; FTD, Frontotemporal Dementia; MCI, Mild Cognitive Impairment; PD, Parkinson's Disease; PSP-S, Progressive Supranuclear Palsy syndrome; NA, Not Available

Age at Onset available in 0/243 (Allon series), 215/329 (Italian series), 0/172 (Northwestern series), 0/302 (Small series), 516/753 (Spanish series), 0/113 (Toronto series), 0/78 (Turkish series), 671/938 (UCSF series), and 162/172 (Other series) patients. Gender available in 243/243 (Allon series), 0/2785 (Gladstone Turkish series), 358/358 (Italian series), 172/172 (Northwestern series), 176/204 (Rush series), 542/542 (Small series), 1173/1174 (Spanish series), 113/113 (Toronto series), 0/444 (Turkish series), 1183/1185 (UCSF series), and 231/231 (Other series) individuals. Ethnicity available in 225/243 (Allon series), 2785/2785 (Gladstone Turkish series), 358/358 (Italian series), 172/172 (Northwestern series), 202/204 (Rush series), 542/542 (Small series), 1174/1174 (Spanish series), 57/113 (Toronto series), 444/444 (Turkish series), 1115/1185 (UCSF series), and 160/231 (Other series) individuals.

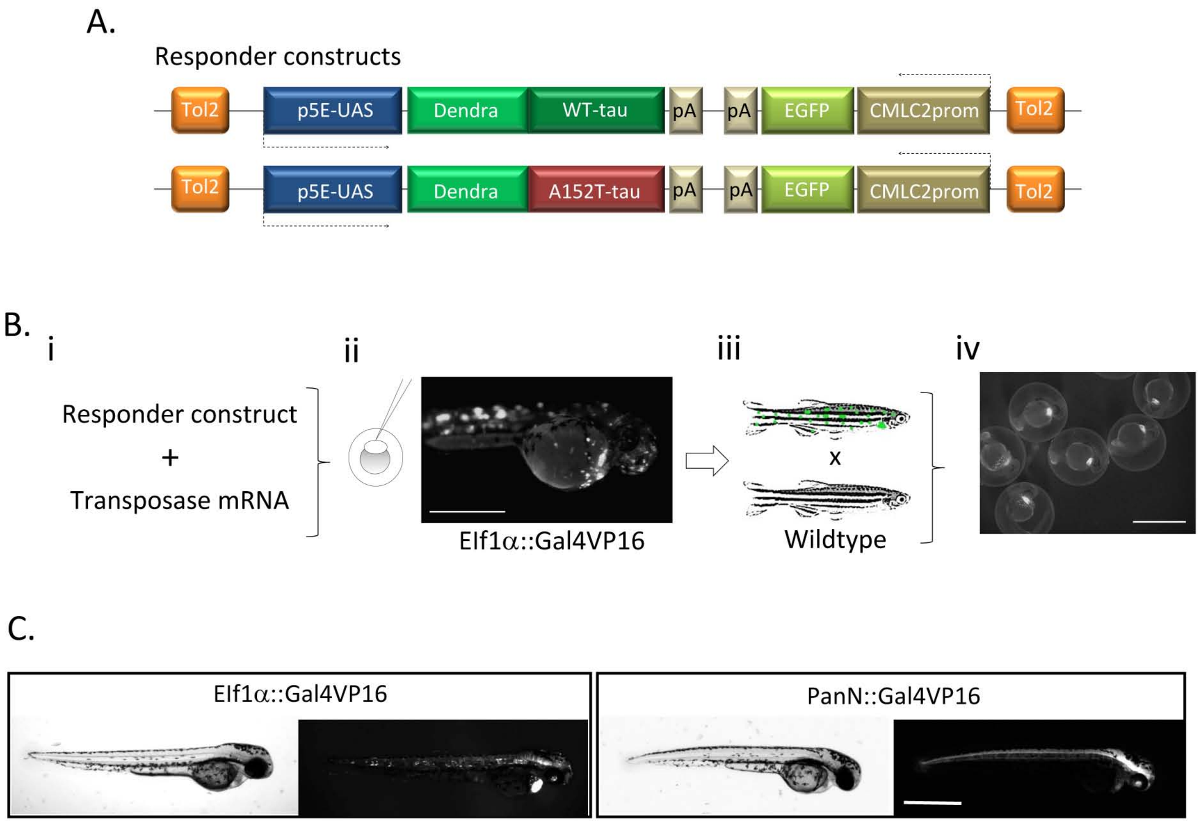

Supplementary Fig.1

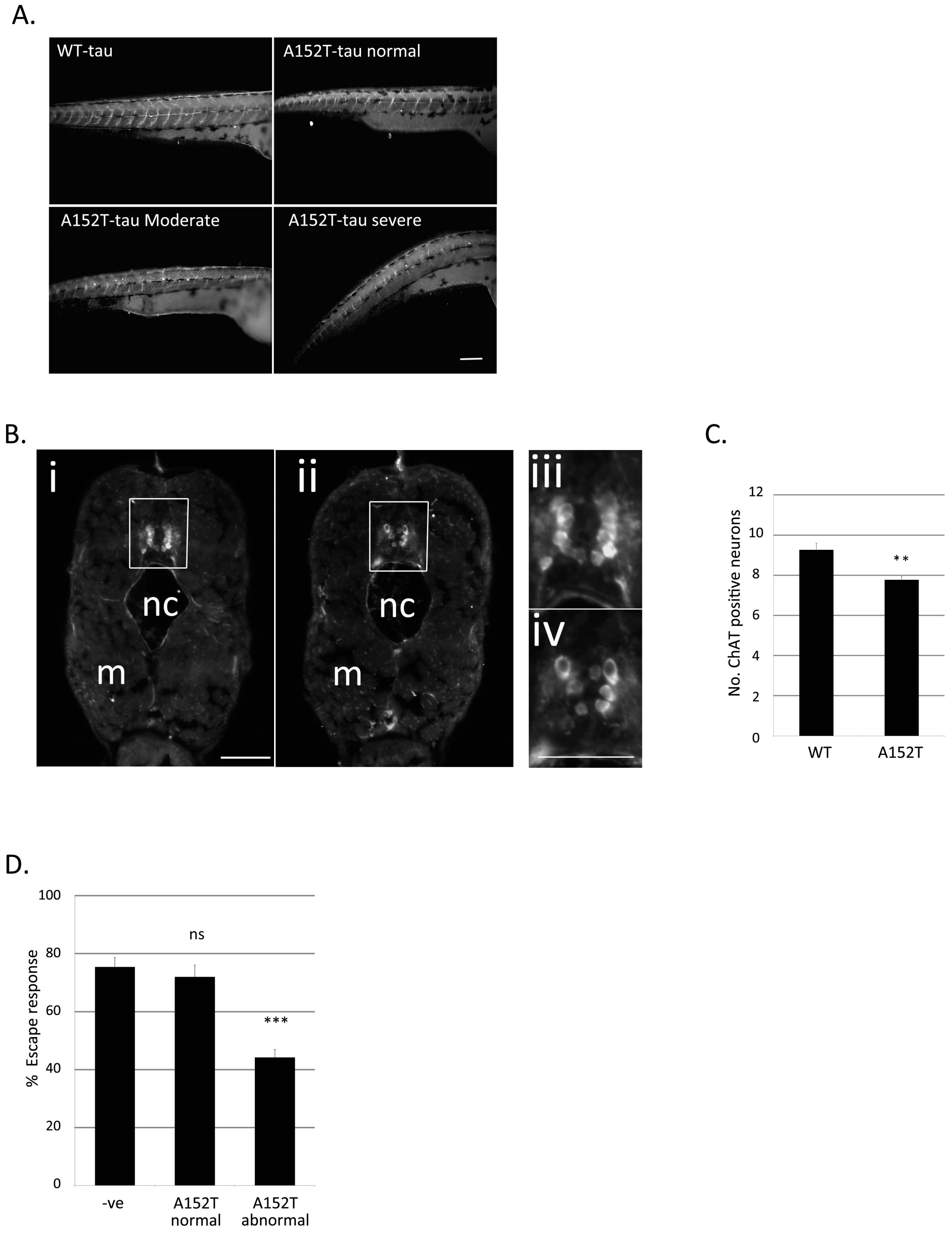

Supplementary Fig.2

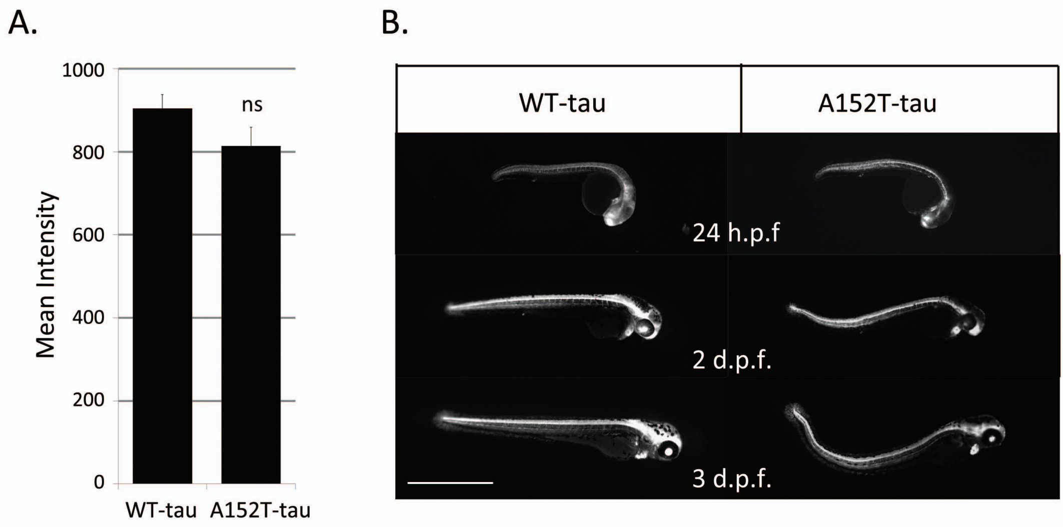
A.
1000
800
600
400
200
0
Mean Intensity
ns
WT-tau
A152T-tau
B.
WT-tau
A152T-tau
24 h.p.f
2 d.p.f.
3 d.p.f.

Supp. Fig 3

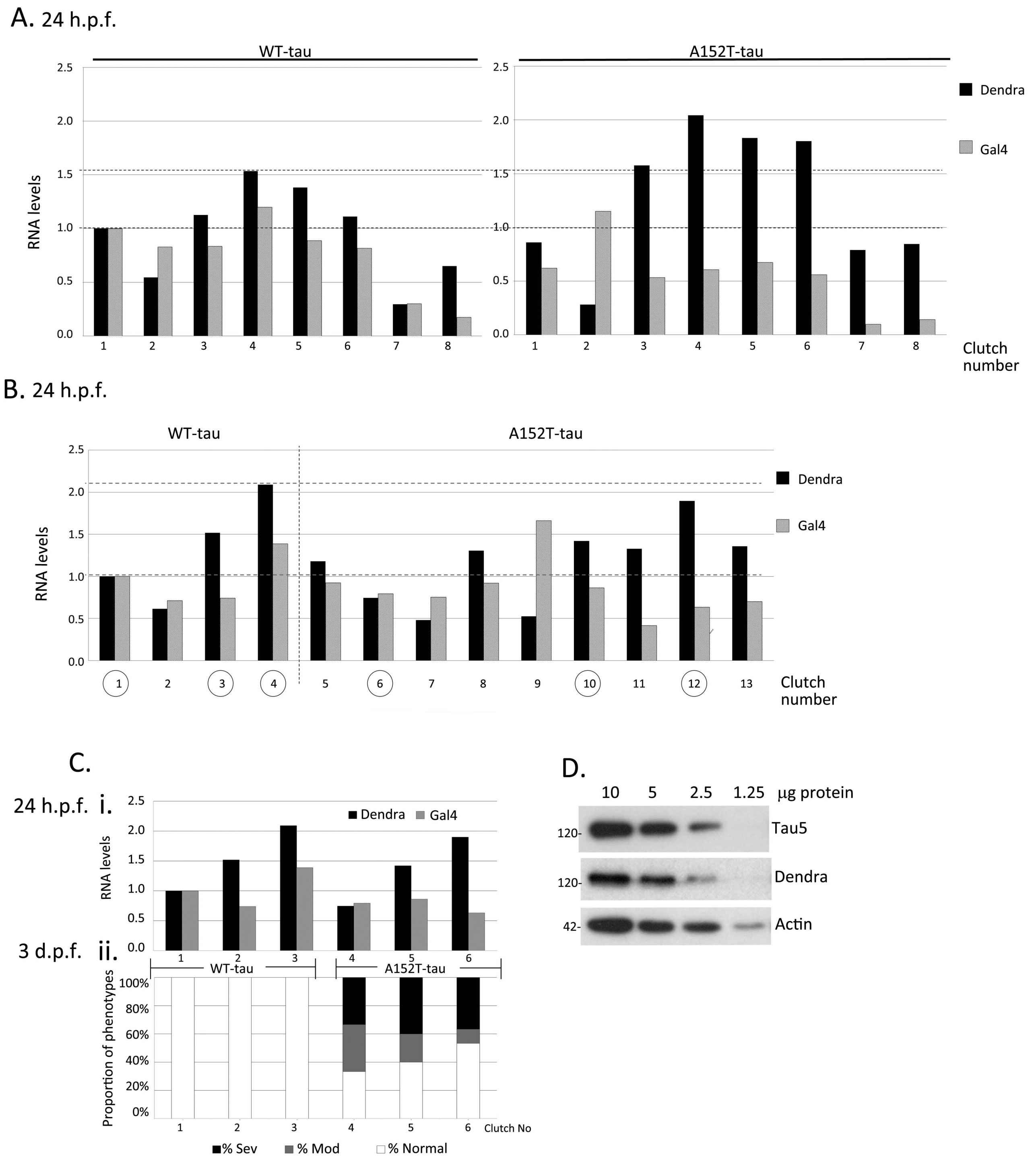
A. 24 h.p.f.
WT-tau
A152T-tau
Dendra
Gal4
RNA levels
2.5
2.0
1.5
1.0
0.5
0.0
1 2 3 4 5 6 7 8
Clutch number
B. 24 h.p.f.
WT-tau
A152T-tau
Dendra
Gal4
RNA levels
1 2 3 4 5 6 7 8 9 10 11 12 13
Clutch number
C.
24 h.p.f. i.
3 d.p.f. ii.
Dendra
Gal4
RNA levels
WT-tau
A152T-tau
Proportion of phenotypes
100%
80%
60%
40%
20%
0%
1 2 3 4 5 6
Clutch No
% Sev
% Mod
% Normal
D.
10 5 2.5 1.25 μg protein
120-
Tau5
120-
Dendra
42-
Actin

A.

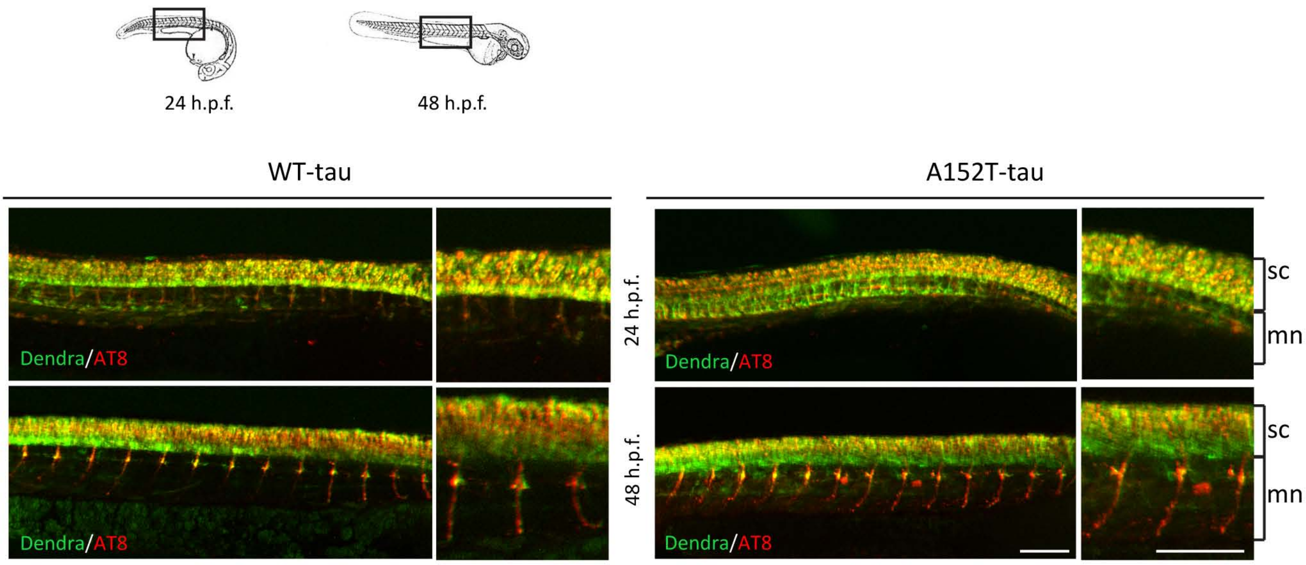
24 h.p.f.
48 h.p.f.
WT-tau
A152T-tau
24 h.p.f.
48 h.p.f.
Dendra/AT8
Dendra/AT8
Dendra/AT8
Dendra/AT8
sc
mn
sc
mn

Supp. Fig 5

A.

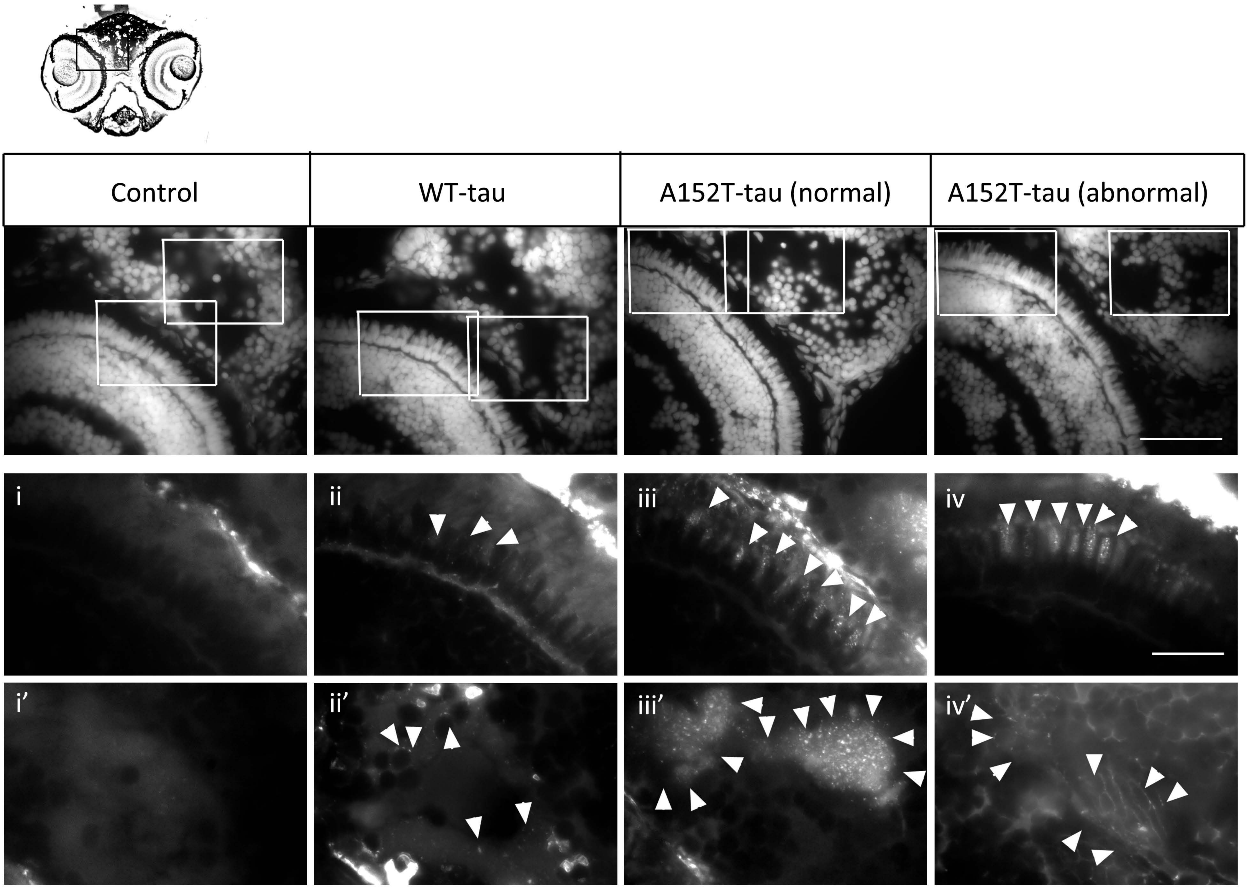
Control
WT-tau
A152T-tau (normal)
A152T-tau (abnormal)
DAPI
ThioS-Retina
ThioS-Brain
i
ii
iii
iv
i'
ii'
iii'
iv'

Suppl Fig.6

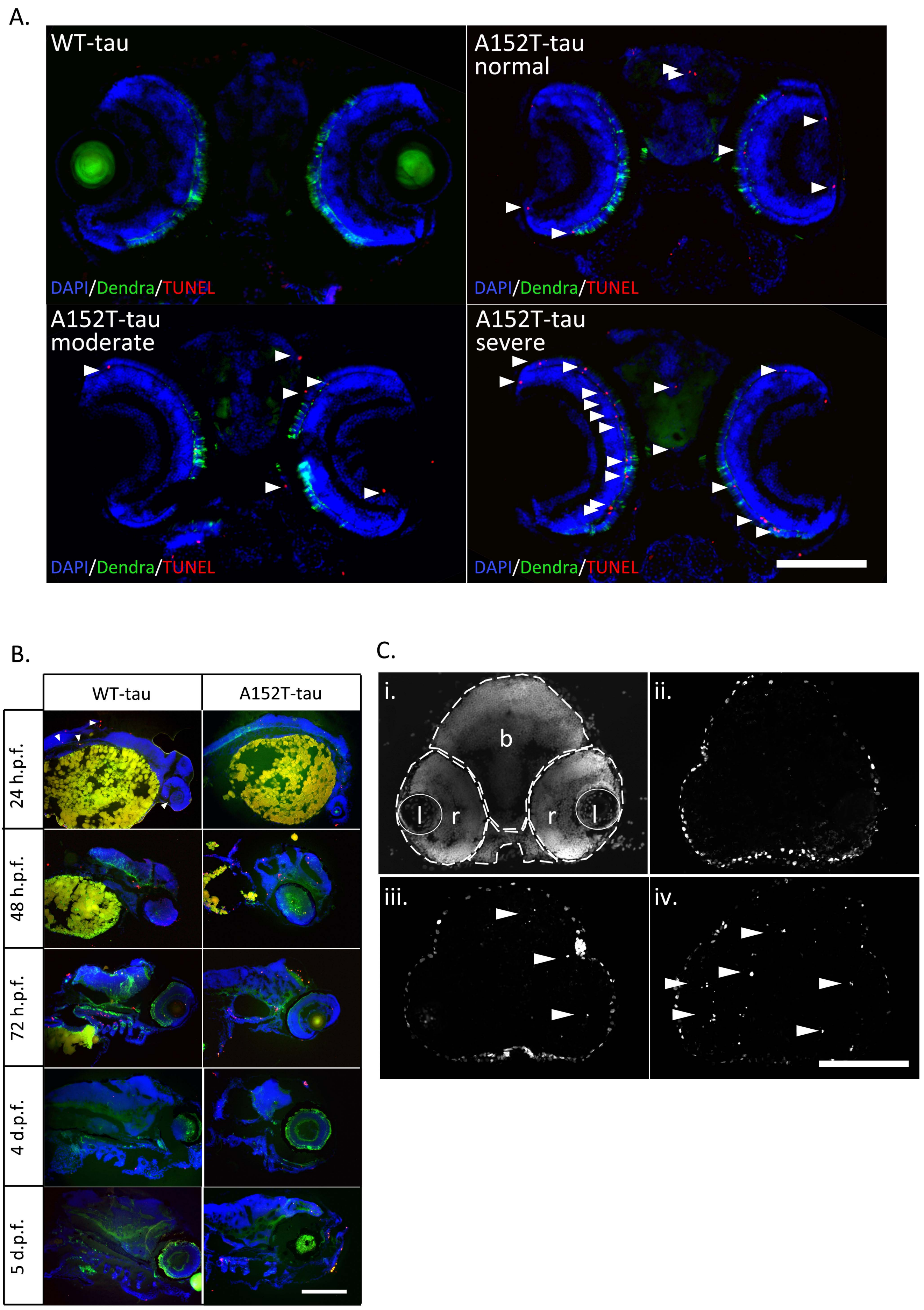

Supplementary Fig.7

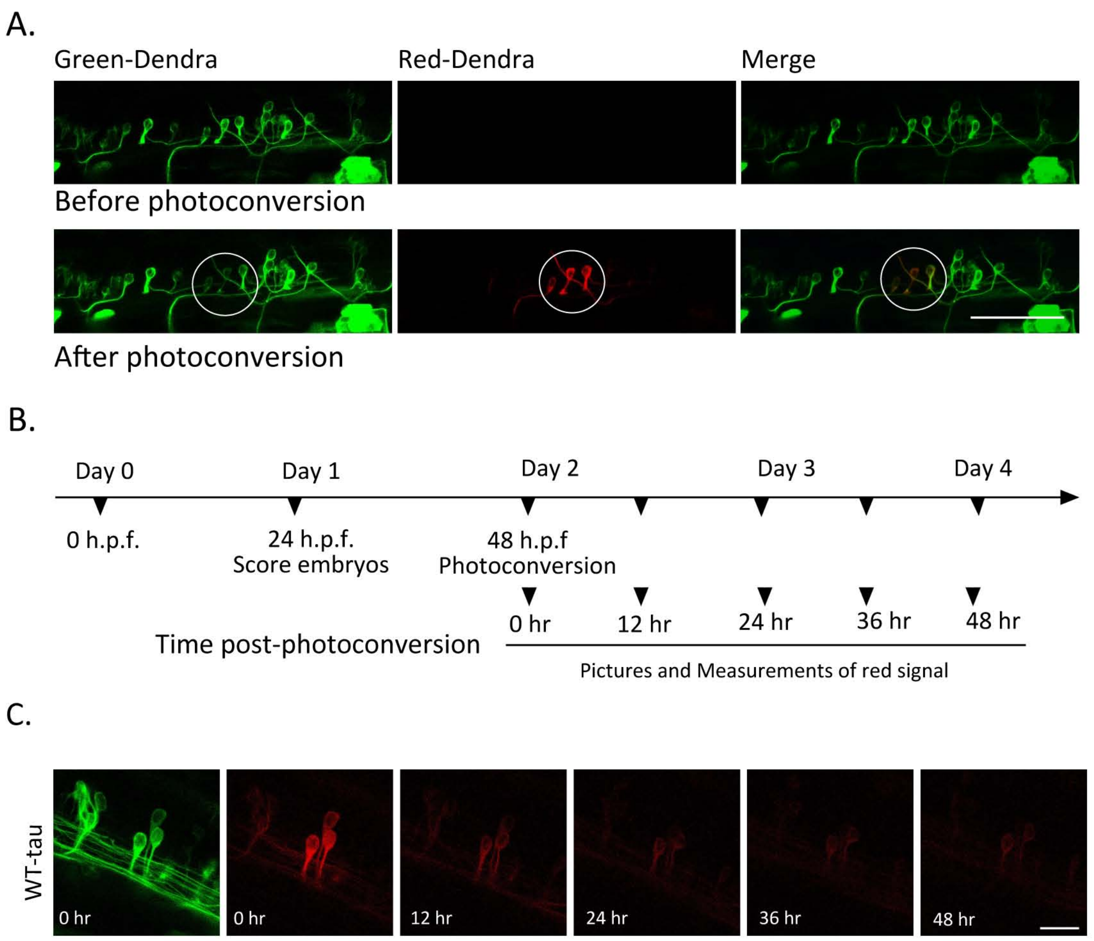
A.
Green-Dendra
Red-Dendra
Merge
Before photoconversion
After photoconversion
B.
Day 0
Day 1
Day 2
Day 3
Day 4
0 h.p.f.
24 h.p.f.
Score embryos
48 h.p.f
Photoconversion
Time post-photoconversion
0 hr
12 hr
24 hr
36 hr
48 hr
Pictures and Measurements of red signal
C.
WT-tau
0 hr
0 hr
12 hr
24 hr
36 hr
48 hr

Supp. Fig 8
